# Wheat Encodes Small, Secreted Proteins That Contribute to Resistance to Septoria Tritici Blotch

**DOI:** 10.3389/fgene.2020.00469

**Published:** 2020-05-12

**Authors:** Binbin Zhou, Harriet R. Benbow, Ciarán J. Brennan, Chanemougasoundharam Arunachalam, Sujit J. Karki, Ewen Mullins, Angela Feechan, James I. Burke, Fiona M. Doohan

**Affiliations:** ^1^UCD School of Biology and Environmental Science, UCD Earth Institute, UCD O’Brien Centre for Science (East), University College Dublin, Dublin, Ireland; ^2^UCD School of Agriculture and Food Science, University College Dublin, Dublin, Ireland; ^3^Department of Crop Science, Teagasc, Carlow, Ireland

**Keywords:** Septoria tritici blotch (STB), *Zymoseptoria tritici*, small secreted proteins (SSPs), wheat disease resistance, apoplastic proteins, protein secretion

## Abstract

During plant–pathogen interactions, pathogens secrete many rapidly evolving, small secreted proteins (SSPs) that can modify plant defense and permit pathogens to colonize plant tissue. The fungal pathogen *Zymoseptoria tritici* is the causal agent of Septoria tritici blotch (STB), one of the most important foliar diseases of wheat, globally. *Z. tritici* is a strictly apoplastic pathogen that can secrete numerous proteins into the apoplast of wheat leaves to promote infection. We sought to determine if, during STB infection, wheat also secretes small proteins into the apoplast to mediate the recognition of pathogen proteins and/or induce defense responses. To explore this, we developed an SSP-discovery pipeline to identify small, secreted proteins from wheat genomic data. Using this pipeline, we identified 6,998 SSPs, representing 2.3% of all proteins encoded by the wheat genome. We then mined a microarray dataset, detailing a resistant and susceptible host response to STB, and identified 141 *Z. tritici*- responsive SSPs, representing 4.7% of all proteins encoded by *Z. tritici* – responsive genes. We demonstrate that a subset of these SSPs have a functional signal peptide and can interact with *Z. tritici* SSPs. Transiently silencing two of these wheat SSPs using virus-induced gene silencing (VIGS) shows an increase in susceptibility to STB, confirming their role in defense against *Z. tritici*.

## Introduction

One of the most economically important species in the plant kingdom is bread wheat, *Triticum aestivum*. Wheat dominates the European arable sector, with ∼150 million tons of wheat grown in the European Union annually (FAOSTAT 2019). While yields are generally high across the EU, wheat production is threatened by a range of pests and pathogens. One of the most important of these is Septoria tritici blotch, a foliar disease caused by the pathogenic fungus *Zymoseptoria tritici* (*Z. tritici*) (O’Driscoll et al., 2014). *Z. tritici* is a strictly apoplastic fungus, and is a host-specific pathogen of wheat. The high selection pressure within intensive agricultural systems [high fungicide usage and dense planting of STB-resistant varieties (Fones and Gurr, 2015)], combined with rapid evolution of the pathogen (Dooley, 2015), has led to the widespread occurrence of *Z. tritici* populations that are resistant to fungicides, or can overcome resistance genes deployed in elite cultivars, or both (Cools and Fraaije, 2013; McDonald and Stukenbrock, 2016; Heick et al., 2017). There are two main phases of STB disease: the symptomless latent phase, during which hyphae of *Z. tritici* enter the leaf tissue via the stomata and begin to colonize the substomatal cavity (Kema et al., 1996), and the subsequent necrotrophic phase. The symptomless phase lasts ∼12 days (dependent on wheat cultivar, *Z. tritici* isolate and environmental conditions) (Hehir et al., 2018), after which the fungus switches to a necrotrophic feeding habit and host tissue begins to die (Keon et al., 2007).

Plants have evolved a multi-layered immune system to recognize and defend themselves against invading pathogens such as *Z. tritici* (Jones and Dangl, 2006). The first layer of plant immunity is pathogen-associated molecular pattern (PAMP)-triggered immunity (PTI). There is a growing body of evidence demonstrating that the apoplast, i.e., the space outside of the plasma membrane, serves as the front-line between the plant host and invading pathogens, and is spatially significant for PTI (Jashni et al., 2015; Wang and Wang, 2018; Schellenberger et al., 2019). Immune receptors on the plant cell surface [known as pattern-recognition receptors (PRRs)], typically with an external binding, lectin or lysin-motif (LysM) domain, play determinant roles during infection by detecting PAMPs; for example the Chitin Elicitor Binding Protein (CEBiP) and Chitin Elicitor Receptor Kinase1 (CERK1), which can recognize the fungal PAMP chitin in *Arabidopsis thaliana* (Miya et al., 2007; Desaki et al., 2018). These receptors activate downstream plant defense responses [encoded by pathogenesis-related (PR) genes], such as the production of reactive oxygen species (ROS), the activation of transcription factors, and the secretion of various pathogenesis-related (PR) proteins into the apoplast that can: hydrolyse glucans, chitin and polypeptides (Tian et al., 2004; Ilyas et al., 2015; Jashni et al., 2015; Ali et al., 2018), inhibit pathogen-secreted enzymes (Kim et al., 2009; Jashni et al., 2015; Rustgi et al., 2018), and phytochemically inhibit pathogen growth (Wirthmueller et al., 2013).

While PRRs recognize and play an important role in resistance to most non-adapted microbes, known as basal resistance (Couto and Zipfel, 2016), when adapted to their host, pathogens can deploy small secreted proteins (SPPs) that act as effectors to suppress or block PTI-induced defense pathways (Block et al., 2014). Hundreds of candidate *Z. tritici* effector genes have been identified via comparative genomics and transcriptomic analyses (Yang et al., 2013; Mirzadi Gohari, 2015; Rudd et al., 2015; Palma-Guerrero et al., 2016; Kettles et al., 2017; Plissonneau et al., 2018). Pathogen effectors are deployed in a spatial and time-dependant manner, depending on the stage of infection. In pathogenic bacteria, effectors are secreted directly out of bacterial cells and/or into the plant cells via multiple secretion systems. For example, the *Pseudomonas syringae* effector HopAO1 and *Ralstonia solanacearum* effector PopP2 are secreted directly into *A. thaliana* plant cells via the bacterial type-III secretion system, and suppress immune responses by targeting receptor kinases and multiple WRKY transcription factors (Macho et al., 2014; Le Roux et al., 2015). In fungi and oomycetes, the effectors are secreted inside (cytoplasmic) or outside (apoplastic) plant cells via the general secretory pathway and through various feeding and infection structures, such as extracellular hyphae and haustoria (Petre and Kamoun, 2014; Wang et al., 2017). During *Z. tritici* infection of wheat, effector proteins are secreted in the apoplast of wheat plant cells, such as Mg3LysM (Marshall et al., 2011; Lee et al., 2014), which interferes with chitin-triggered immunity and helps establish the disease during the latent phase of infection. Although the causes for the rapid switch to necrotrophy in the *Z. tritici* life cycle are largely unknown, several *Z. tritici* effectors have been implicated in initiating the necrotrophic phase, such as MgNLP, ZtNIP1, and ZtNIP2 (Marshall et al., 2011; Ben et al., 2015).

In response to the secretion of effectors, plants have developed a second layer of immunity, in which host nuclear-binding leucine-rich repeat (NLR) proteins, typically characterized by an extracellular leucine-rich repeat (LRR) domain (Chiang and Coaker, 2015), recognize pathogen effectors. Recognition of effectors, leading to ETI, can elicit a hypersensitive response, often associated with salicylic acid (SA) signaling and systemic acquired resistance (SAR) (Kombrink and Schmelzer, 2001). Additionally, plant small secreted proteins have also been reported to play key roles in plant immunity (Lanver et al., 2017; Ziemann et al., 2018; Segonzac and Monaghan, 2019). The *A. thaliana* protein AtPep1 is a 23 AA long peptide that enhances plant resistance to various pathogens, including the bacterium *P. syringae*, the fungus *Botrytis cinerea*, and the oomycete *Phytophthora infestans* (Huffaker et al., 2006; Yamaguchi et al., 2010; Liu et al., 2013). In maize (*Zea mays*), the ortholog of AtPep1 (ZmPep1) was demonstrated to activate the production of jasmonic acid and induce multiple defense pathways to enhance resistance against the fungal pathogens *Cochliobolus heterostrophus* and *Colletotrichum graminicola* (Huffaker et al., 2011). Additionally in *Z. mays*, a 17 AA peptide, termed *Z. mays* immune signaling peptide 1 (Zip1), is a functional elicitor of SA signaling in maize (Ziemann et al., 2018). In the case of the wheat - *Z. tritici* interaction, wheat can secrete β-1,3-glucanase into the apoplast, which cleaves β-1,3-glucan in the *Z. tritici* cell wall to prevent colonization of *Z. tritici* (Shetty et al., 2009).

These findings have demonstrated that plant secreted proteins play significant roles in apoplastic immunity in plant–pathogen interactions, and that plant-encoded SSPs may be an important reservoir of potential STB-resistance genes for wheat. Using features typical of small secreted proteins, such as a protein length ≤ 250 amino acids and a secretion signal of an N-terminal signal peptide, we investigated the small secretome of wheat, to identify small secreted proteins from that may play a role in the wheat – *Z. tritici* interaction, and may interact with fungal SSPs that are also present in the apoplast during infection. The aims of this study were to determine: (1) if wheat-encoded SSPs are regulated during wheat-*Z. tritici* interactions, (2) whether some SSPs might be able to enhance wheat resistance to *Z. tritici* and (3) if yes, how molecular mechanisms for SSPs contribute to wheat resistance.

## Materials and Methods

### Plant and Fungal Material

Wheat (*T. aestivum*) cultivars (cvs.) Stigg and Gallant were used in this study. The cv. Stigg [Pedigree: (BISCAY/LW-96-2930//TANKER)] is resistant to STB disease (Hehir et al., 2018) and cv. Gallant (Pedigree: TJB-268-175/HOBBIT) is susceptible to STB disease (Orton and Brown, 2016). Wheat seeds were incubated at 4°C for 5 days then subsequently transferred to a dark 19°C growth room for 3 days. Germinated seeds were transferred to 2 L trays filled with John Innes Compost No. 2 soil (Westland Horticulture, United Kingdom). Plants were grown under controlled conditions at 19°C with a 15/9 h light/dark cycle and the relative humidity was maintained at 80% using a Humidisk 10 humidifier (Carel, Italy). *Nicotiana benthamiana* seeds were incubated at 4°C for 3 days in a cold room. Then the seeds were transferred to a growth chamber at 22°C (day) to 19°C (night) with a 16/8 h light/dark for 5 weeks before infiltration for all experiments.

The *Z. tritici* isolate used in this study was a field isolate collected from the wheat cv. Cordiale in Cork, Ireland hereafter referred to as ‘Cork Cordiale 4.’ Glycerol stocks were provided by Dr. Thomas Welch (Teagasc, Crops Research Centre, Carlow, Ireland). *Z. tritici* was cultured by inoculating YPDA (10 g Yeast extract, 20 g Bacteriological peptone, 20 g D-Glucose, and 15 g Agar in 1 L water) plates with 50 μl of the glycerol stock to generate conidia. The petri dishes were transferred to a near-ultraviolet light incubator for 7 days at 20°C with a 12:12 h light/dark cycle. Plates were flooded with 3 mL sterile water and scraped with a sterile spreader to collect the *Z. tritici* spores (pycnidiospores). Spores were filtered through sterile cheesecloth and the concentrations were measured using a Glasstic hemocytometer (Kova International, United States). The final spore concentration was adjusted to 1 × 10^6^ per ml and 0.02% Tween20 (Fisher Bioreagents, United States).

### Identification and Characterization of Wheat Small Secreted Proteins

**FIGURE 1:**
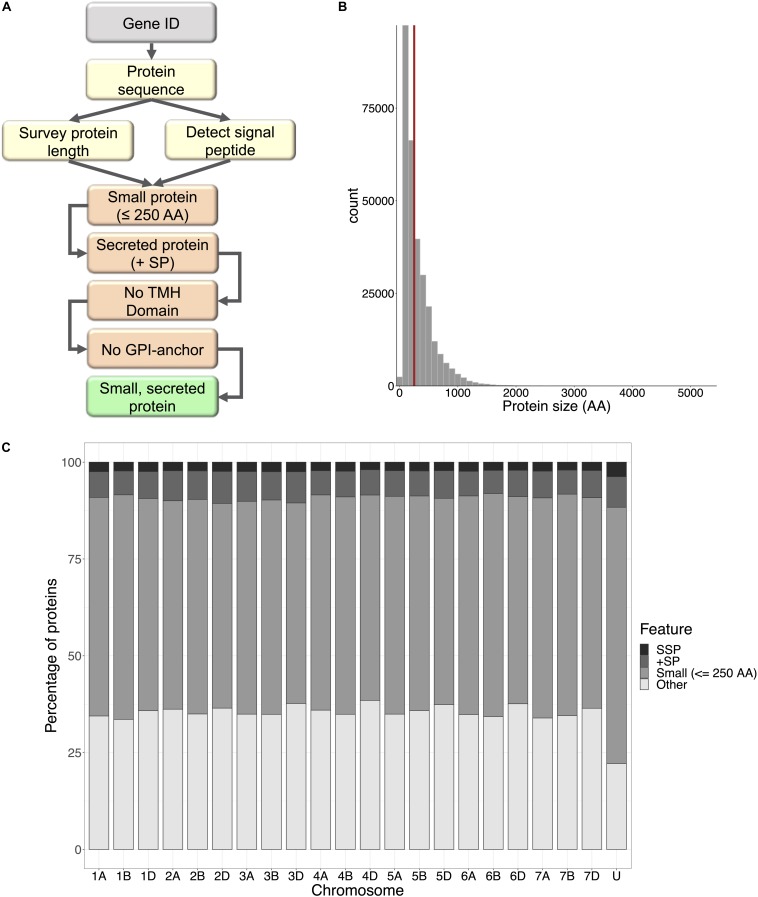
Small secreted proteins of wheat. **(A)** The small secreted protein (SSP) discovery pipeline, written in Bash command and R languages. Protein sequences are retrieved for query gene IDs, surveyed for length and SignalP v5.0 was used to detect presence of a signal peptide. Small proteins with a signal peptide were filtered for transmembrane helix domains (TMH) and GPI-anchors. The resulting proteins were designated SSPs. **(B)** Frequency distribution of protein size for all proteins encoded within the wheat genome. The cut-off size for SSPs (250 amino acids) is shown by the red line; 58% of all wheat proteins were ≤250 AA. **(C)** The percentage of small proteins, proteins with a signal peptide, and SSPs with no TMH domains and GPI-anchors encoded by genes on each chromosome of the wheat genome.

A bioinformatics pipeline was developed to automate the identification of wheat small, secreted proteins (TaSSPs), written in the Bash-command and R languages (Figure 1A). Briefly, the script takes gene IDs as an input and retrieves their corresponding protein sequences from the IWGSC refseq V1.1 protein annotation^1^ (IWGSC, 2018), using the SAMtools fasta index function (Li et al., 2009; Li, 2011). The length of each query protein was retrieved, and the standalone SignalP V5.0 software (Armenteros et al., 2019), with default parameters, was used to detect the presence of a signal peptide and the location of their cleavage site in the protein sequences (Armenteros et al., 2019). The pipeline scripts are open access and can be accessed at https://github.com/hbenbow/SSP_pipeline.git. Using this pipeline, the entire wheat proteome was searched for SSPs, by splitting the reference protein annotation by chromosome and mining each chromosome individually. The resultant set of predicted SSPs was further refined by identifying and removing proteins with any transmembrane helices, [using TMHMM v2.0 (Krogh et al., 2001)], and glycosylphosphatidylinositol (GPI)-anchors [using GPI-SOM (Fankhauser and Maser, 2005)]. *TaSSP* genes were annotated using Blast2GO (Conesa et al., 2005), and a Fisher’s enrichment test was carried out between the *TaSSP* set and the whole genome to test if any GO terms were significantly enriched in the *TaSSP* set [note; only high-confidence *TaSSP* genes (i.e., 4,532 of the total 6,998) were included in this analysis, as only high confidence gene annotations were present in the reference Blast2GO annotation]. To further characterize the small secreted proteins, the signal peptide sequence was cut from the sequence FASTA file using the Bedtools getfasta algorithm (Quinlan and Hall, 2010), using a.BED file of coordinates from 1:*n*, where *n* = the cleavage site position defined by SignalP. The MEME-suite (Bailey et al., 2009) was used to identify motifs in the signal peptides, using the options -nmotifs 10 to find the top 10 motifs in the set of signal peptide sequences. Following MEME, MAST (Bailey and Gribskov, 1998) was used to align the motifs back to the sequences and identify which sequences contained which motifs. To identify signal peptide sequences that were similar to each other, the sequences were clustered with CD-HIT (Li et al., 2001), where sequences with >90% similarity to each other were clustered into groups. The distribution of clusters and cluster size was reported using the Perl script plot_len.pl from https://github.com/weizhongli/cdhit.

### Identification and Validation of *Z. tritici*-Responsive SSPs

*Zymoseptoria tritici*-responsive SSPs were identified using the differentially expressed probe set from Brennan et al. (2020, in press); a microarray study, which assessed the transcriptome responses of winter wheat cvs. Stigg and Gallant to *Z. tritici* (isolate IPO323) at 4, 8, and 12 days post-inoculation (dpi), available at https://doi.org/10.6084/m9.figshare.11882601.v1. Microarray probe sequences were retrieved from Affymetrix^2^. Probe sequences for every differentially expressed probe in each cultivar × timepoint combination were BLASTn searched against the IWGSC v1.1 reference CDS annotation (IWGSC, 2018) using BLAST+. As the microarray probes could potentially hybridize to all three homoeologues of each wheat gene, a one-to-one search algorithm was not appropriate for identifying the full gene sequence of each microarray probe. Therefore, bespoke Bash and R scripts were created to identify the top three IWGSC hits for each microarray probe. The probe sequences were used as the query and the IWGSC reference was used as the search subject. The BLASTn short sequence algorithm was used with the parameters -max_target_seqs 1, -max_hsps 3 and -task blastn-short, to return a maximum of 3 high-scoring pairs. The BLASTn results were returned in tabular format (-outfmt 6). The output file was sorted first by query ID, then by (in this order): bitscore (descending), *E*-value (ascending) and percentage identity (descending). From this sorted file, the top three hits for each query sequence were retained. These scripts are open access and can be accessed at https://github.com/hbenbow/SSP_pipeline.git. *Z. tritici-*responsive genes were cross-referenced against the list of SSPs. To choose candidate *Z. tritici* -responsive SSPs, we focused on SSP genes with a high fold-change in cv. Stigg (resistant) compared to cv. Gallant (susceptible), and candidates were chosen for cloning and further study. *In silico* analysis of these genes was done using a BLASTx search to the NCBI non-redundant nucleotide database, using default parameters, and InterProScan. Both of these were perform as part of the OmicsBox desktop application.

Expression of candidate SSP genes was validated by qRT-PCR as per Brennan et al. (2020). Plants of cvs. Stigg and Gallant were grown as stated above, and at growth stage 21 (Zadocks et al., 1974), the third leaf was spray inoculated with 1 ml (1e^6^ spores) *Z. tritici* on both the adaxial and abaxial surface using a Hozelock 0.5 L hand-held mist sprayer (Hozelock LTD., United Kingdom). Control plants were inoculated with a solution of 0.02% Tween20. A total of three independent trials were conducted, each with four plants (2 per pot) per time point per cultivar per treatment. At 4, 8, and 12 dpi, the entire third leaf was excised and flash frozen in liquid nitrogen for RNA extraction.

### RNA Extraction and cDNA Synthesis

Leaf tissue was ground in liquid nitrogen in a sterile mortar and pestle. Total RNA was isolated using the RNeasy Plant Mini Kit (Qiagen, Germany) following the manufacturer’s recommendations. gDNA was removed from RNA extraction samples using TURBO DNA-free^TM^ Kit (Ambion, United States) in accordance with the manufacturer’s protocol. RNA quality and integrity were checked using an ND-1000 Spectrophotometer NanoDrop (Thermo Scientific, United States) and it was visualized on a 1.5% agarose gel. DNA removal was validated by PCR using Glyceraldehyde-3-phosphate dehydrogenase (GAPDH)-specific primers, which span an intron (Supplementary Table S1). Each PCR reaction contained 0.125 μl Ex Taq^TM^, 2.5 μl 10X Ex Taq Buffer, 2 μl dNTP mixture, 2 μl treated RNA sample (or 2 μl gDNA 50 ng/μl serving as the positive control), 2 μl 5 μM Primer in 25 μl reaction volume, with following conditions: 1 cycle of 30 s at 98°C; 40 cycles of 5 s at 98°C and 20 s at 60°C; and a final cycle of 2 min at 72°C. PCR products were visualized using 1.5% agarose gel electrophoresis. Reverse transcription of total RNA was performed using SuperScript II Reverse Transcriptase (Invitrogen, United States) following the manufacturer’s recommendations. Two cDNA samples were synthesized from each RNA sample.

### Quantitative Real-Time PCR (qRT-PCR) Analysis

Quantitative real-time PCR (qRT-PCR) analysis was conducted using the Stratagene Mx3000TM Real-Time PCR (Stratagene, United States). Each reaction was performed with 1.25 μL of a 1:5 (V/V) dilution of cDNA, 0.2 μM of each of the primers (Supplementary Table S1) and 1X SYBR Premix Ex Taq (Takara, Japan) in a total reaction volume of 12.5 μL, with following conditions: 1 cycle of 1 min at 95°C; 40 cycles of 5 s at 95°C and 20 s at 60°C; and a final cycle of 1 min at 95°C, 30 s, at 55°C, and 30 s at 95°C for the dissociation curve. All real-time qRT-PCR analyses were conducted in duplicate. Two housekeeping genes were used as reference genes, α-tubulin and Glyceraldehyde phosphate dehydrogenase 2 (GAPDH2) (Supplementary Table S1).

The threshold cycle (Ct) values obtained by real-time RT-PCR were used to calculate the ΔCt values for the formula ΔCt = Ct_(target gene)_ − μ[Ct_(housekeeping genes)_]. Relative expression was calculated using the formula 2^–^^Δ^^*Ct*^ (Livak and Schmittgen, 2001). qRT-PCR was carried out for each of the three independent trials, with 2 reactions per cDNA, and 2 cDNAs per RNA extraction.

### Cloning of Wheat Small Secreted Protein (*TaSSP*) Encoding Genes

The full length *TaSSP* genes were amplified from cDNA produced from *Z. tritici*-infected wheat leaf (cv. Stigg or Gallant) using primers matching the 5′ and 3′ UTR of the *TaSSPs* genes (Supplementary Table S1). The PCR reactions (50 μl) contained 0.25 μl Ex Taq^TM^, 5 μl 10X Ex-Taq Buffer, 4 μl dNTP mixture, 2 μl treated cDNA, 4 μl primer (5 μM), and 3 technical replicates with the following program constituted 98°C for 30 s, 35 cycles of 98°C for 10 s, extension of 68°C for 1 min, with a final extension at 72°C for 5 min. The PCR product was cloned into pDONR207 (Invitrogen, United States) after the 2nd amplification using the attB1 and attB2 primers (Supplementary Table S1) and was subsequently introduced into different expression vectors by the Gateway cloning technology (Invitrogen, United States) for gene function analysis.

### Developing a Sucrose Transport Protein Signal Sequence Trap System and Testing of TaSSPs Secretion

A yeast sucrose transport protein *SUC2* signal sequence trap system was developed and used to determine whether TaSSP proteins were secreted (the schematic diagram of the yeast secretion assay is shown in Supplementary Figure S1). The sucrose transport protein *SUC2* gene of *Saccharomyces cerevisiae* strain SEY6210 (ATCC: The Global Bioresource Center) was replaced by the tryptophan synthesis (*Trp1*) gene via homologous recombination (Horecka and Davis, 2014) using primers POP-IN-U2-F, POP-IN-D2-R, Pop-Trp-U2-F and Pop-Trp-D2-R (Supplementary Table S1). The *suc2* mutant yeast cells were selected on synthetic Trp dropout (-Trp) yeast media (Takara, Japan). To construct yeast expression vectors for the secretion assay, the full length and truncated (without signal peptide, SUC^22^^–^^511^) *SUC2* genes were cloned into the pGADT7 plasmid (Clontech, United States) using the NEBuilder HiFi DNA Assembly Cloning Kit (NEB, United States) according to the manufacturer’s instructions. A DNA fragment containing Gateway cassette, HA tag, Kex2 cleavage site (TCTCATGGTTCTTTGGATAAAAGAGAGGCTGA) and SUC^22^^–^^511^ gene was synthesized by General Biosystems (United States) and ligated into the pGADT7 plasmid (Clontech, United States) to generate a Gateway compatible vector pGAD-GW-SUC2^22^^–^^511^ for TaSSP protein secretion assays in yeast. All yeast expression vectors sequences for secretion analysis are presented in Supplementary Figure S2. To test the secretion of TaSSPs, the candidate TaSSP genes were cloned into the secretion vector pGAD-GW-SUC2^22^^–^^511^ using Gateway recombination cloning technology (Invitrogen, United States). The *TaSSP* genes were fused to the N-terminus of the SUC^22^^–^^511^ gene. The vectors were transformed into the *suc2* mutant yeast following the yeast transformation protocols (Sigma-Aldrich). Yeast was spread on a synthetic Trp and Leu dropout (-TL) plates with sucrose (10 mM) as the sole carbon source. The Petri dishes were transferred to an incubator at 28°C for 3 days. If TaSSPs were secreted, the positive *suc2* mutant yeast transformants grew on the plate and were visible after 3 days (Plett et al., 2017). Four trials were conducted, in which nine independent yeast clones were grown and three technical reps from each yeast clone were tested. Three biological replicates were conducted per trial and yeast spotting on the media was performed using serial dilutions from an initial OD_600_ of 1.0, 0.1, 0.01, and 0.001, respectively.

### Yeast Two-Hybrid Analysis

The interaction between TaSSP proteins and *Z. tritici* SSPs (ZtSSPs) were assessed via yeast two-hybrid (Y2H) analysis. Twenty-seven non-annotated ZtSSPs were identified by filtering the publicly available secretome dataset from do Amaral et al. (2012) to identify Z. tritici small secreted proteins (ZtSSPs). The candidate sequences were filtered based on the following features: EST support, size (≤315 aa), presence of cysteine residue, presence of signal peptide using SignalP v5.0 (Armenteros et al., 2019) and lack of transmembrane domain predicted by TMHMM v2 (Krogh et al., 2001). Finally, putative secreted proteins with unknown functional conserved domains were selected using NCBI CDD (Conserved Domain Database) and the Pfam database (JGI Protein ID of predicted ZtSSPs is listed in Supplementary Table S2). Truncated *TaSSP* and *ZtSSP* genes (lacking their signal peptides) were amplified by PCR using gene-specific primers (Supplementary Table S1) and cloned into the vector pDONR207 using the Gateway cloning technology (Invitrogen, United States). Both TaSSPs and ZtSSPs were then recombined into bait and prey vectors derived from pGADT7 and pGBKT7 plasmids (Clontech, United States). The bait and prey vectors were transformed into a yeast strain (Y2H Gold, Clontech) and grown on Trp and Leu drop-out medium (-TL) at 28°C for 3 days. The yeast cells carrying both plasmids were selected on Trp/Leu/His/Ade drop-out medium (-TLHA). Three technical replicates were performed per TaSSP-ZtSSP combination. If TaSSPs interacted with ZtSSPs, the yeast can grow on -TLHA plates at approximately 3–7 days. Three trials were performed, in which nine independent yeast clones were grown and divided into three technical replicates to be tested.

### Bimolecular Fluorescence Complementation

Bimolecular fluorescence complementation (BiFC) was used to validate the interactions between TaSSPs and ZtSSPs *in planta*. The relevant pDONR207 vectors encoding *TaSSPs* and *ZtSSPs* used for Y2H were recombined into the BiFC vectors pDEST-VYCE^*GW*^ and pDEST-VYNE^*GW*^ (Gehl et al., 2009) using Gateway cloning technology (Invitrogen, United States). This generated constructs wherein proteins were fused to the YFP C-terminal (YFP^*C*^) or N-terminal fragment (YFP^*N*^). The vectors were then transformed into the *Agrobacterium tumefaciens* strains GV3101 by electroporation. The transformed GV3101 strains were cultured in LB liquid medium containing gentamicin (20 μg/ml), kanamycin (50 μg/ml), and rifampicin (50 μg/ml) at 28°C overnight. *A. tumefaciens* was harvested by centrifuge at 4000 rpm for 10 min and washed once with distilled water. The *A. tumefaciens* cells were resuspended in infiltration buffer (10 mM MES pH 5.6, 10 mM MgCl_2_, and 150 μM acetosyringone) to an OD_600_ = 0.5 and incubated in the dark for 2 h at room temperature. The leaves of 5 weeks old *N. benthamiana* plants were infiltrated using a 1 ml needleless syringe. Epidermal cells of leaves were assayed for YFP fluorescence using a Confocal Laser Scanning Microscope (Olympus fluoview FV1000) at 2 days post-infiltration. YFP fluorescence was excited at 515 nm and detected in the range between 530 and 630 nm. Three trials were conducted and within each trial, three independent leaves were analyzed per TaSSP-ZtSSP combination.

### *Agrobacterium*-Mediated Expression of ZtSSPs

It is reported that some ZtSSPs can induce cell death in *N. benthamiana* leaves (Kettles et al., 2017). The ZtSSPs that interacted with TaSPPs were cloned into a high-level expression vector pEAQ-HT-DEST3 (Sainsbury et al., 2009) using Gateway cloning technology (Invitrogen, United States). The constructs were transformed into *A. tumefaciens* strains GV3101 by electroporation. Five week old *N. benthamiana* plants were infiltrated as described by Kettles et al. (2017). The only modification was that the GV3101 was finally resuspended at OD_600_ = 1.0 before infiltration. The infiltrated leaves were observed for 10 days to check for cell death. Four independent leaves were analyzed per ZtSSP per trial, and three trials were conducted in total. As a negative control, GFP was infiltrated into four independent leaves per trial. To test if infiltration of co-expressed ZtSSP-TaSSP combinations affected the cell death phenotyping in *N. benthamiana* leaves, co-expressed proteins were infiltrated into *N. benthamiana* leaves as above, and six biological replicates (from three independent plants) were conducted.

### Virus-Induced Gene Silencing (VIGS)

Virus-induced gene silencing (VIGS) was used to determine the impact of *TaSSP* genes on STB disease, based on the Barley Stripe Mosaic Virus (BSMV) method (Scofield et al., 2005; Gunupuru et al., 2015). Two gene fragments were used for VIGS of TaSSP6 and TaSSP7 genes and these were amplified from the CDS of TaSSP6 and TaSSP7 (VIGS primer sets are listed in Supplementary Table S1). VIGS target sequences were chosen to preferentially silence all three (A, B, and D genome) homoeologues (where present) using the publicly available online Wheat Ensembl database^3^. The PCR amplicons of silencing fragments were digested and ligated into BSMV-γ vectors using *Not*I/*Pac*I (NEB, United States). They were named BSMV:TaSSP6-V1, BSMV:TaSSP6-V2, BSMV:TaSSP7-V1, and BSMV:TaSSP7-V2. Inserted gene fragments were confirmed by sequencing. In addition, a BSMV-γ vector with silencing fragments for phytoene desaturase (PDS) was used as a positive control and a BSMV-γ empty vector as a negative control. Plasmid linearization, *in vitro* transcription of RNA, and flag leaf inoculation with 1:1:1 mixtures of the *in vitro* transcripts of BSMV α, β, and γ RNA were done as previously described (Scofield et al., 2005). Plants were placed in low light conditions overnight and allowed to recover from mechanical stress; thereafter plants were returned to normal growth conditions. The *Z. tritici* inoculation was applied to the third and fourth leaves of wheat plants at 7 days post-VIGS constructs inoculation. The third leaf of each plant was taken for qRT-PCR validation at 8 days after *Z. tritici* inoculation and the fourth leaf was used for subsequent phenotyping. STB disease severity was assessed by scoring the percentage of leaf area bearing necrosis at 21 dpi. The leaves were then excised from the plant and placed in 100% humidity to promote pycnidia growth. For the VIGS experiment, each trial included 12 plants per treatment combination and the trial was replicated three times.

### Statistical Analysis

All statistical analyses were performed in R v3.5.2. Data were checked for normality using a Shapiro–Wilk test. To adjust for variation between the trials for the gene expression time course, relative expression was adjusted to a percentage of relative gene expression in control plants of cv. Gallant at 4 DPI. Data were transformed using Johnson transformation and One-way ANOVA was used to calculate differences in gene expression between Cultivar + Treatment + Timepoint combinations. Tukey’s HSD test was used for multiple comparisons of means. A Kruskal–Wallis test was used to analyze VIGS phenotype data with Dunn’s *post hoc* test. VIGS qRT-PCR data for SSP6 were analyzed using a Kruskal–Wallis test and means were compared using Dunn’s test, and SSP7 data were analyzed using One-way ANOVA with Tukey’s HSD *post hoc* test.

## Results

### Identification of Wheat Small Secreted Proteins (TaSSPs)

**FIGURE 2:**
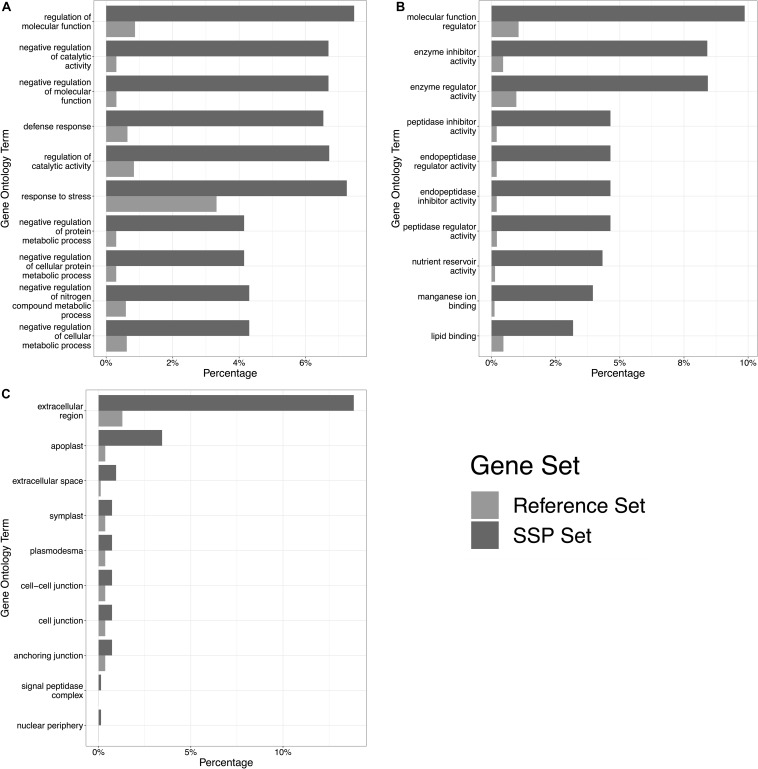
The top 10 significantly enriched gene ontology (GO) terms in the *TaSSP* gene set. Panel **(A)** shows overrepresented biological processes, **(B)** shows overrepresented molecular functions, and **(C)** shows overrepresented cellular components. High-confidence genes encoding for predicted TaSSPs were tested against the complete wheat gene annotation, and 177 GO terms with a significant over-representation in the TaSSP set were retrieved. The percentage of *TaSSP*s in each GO category are shown by dark gray bars (“SSP Set” in the legend), and the percentage of genes within the whole wheat reference in each GO category is shown by pale gray bars (“Reference Set” in the legend).

From across the wheat protein reference (298,774 proteins), the mean protein length was 311 AA, and the median was 218 AA (Figure 1B). The shortest proteins were 12 AA long, and the longest was 5,360 AA. We identified 166,086 small (≤250 AA) proteins, 20,763 proteins with a predicted signal peptide, and 8,467 (2.8% of the wheat proteome) proteins that were small and had a signal peptide (Figure 1C). From this set, 1,460 proteins had a predicted GPI-anchor, 12 contained more than one transmembrane helix (TMH) domain, and 3 had both GPI-anchor and TMH. This left a total of 6,998 unique wheat proteins that were classified as ‘small, secreted proteins,’ representing 2.3% of the wheat protein annotation. The SSPs are available in Supplementary File 2. The percentage of SSPs attributed to the 21 wheat chromosomes ranged from 1.9–2.4%, and 3.7% were attributed to chromosome ‘Un’ (a chromosome reference that contains assembled sequence contigs that have not been unambiguously assigned to a chromosome) (Table 1). Of the annotated *TaSSP* genes, the most dominant biological process was the negative regulation of peptidase activity. The top biological processes also included the defense response, the negative regulation of catalytic activity, and lipid transport. The most common molecular functions were nutrient reservoir activity, manganese binding activity and enzyme inhibitor activity. The most common cellular component of the *TaSSPs* was the extracellular region, followed by the membrane and the apoplast. The Fisher’s enrichment test revealed that 119 biological processes, 46 molecular functions, and 12 cellular components were significantly (FDR < 0.05) enriched (over-represented) in the *TaSSP* set, compared to the full wheat gene reference set. The most over-represented GO terms included the regulation of molecular functions, the negative regulation of catalytic activity, enzyme inhibitory activity, the defense response, peptidase inhibitory activity, the extracellular region and the apoplast, among others (Figure 2).

**TABLE 1:** The number of small, secreted proteins (SSPs) in the wheat genome.

Chromosome	Whole genome			Filtered		SSPs^e^
		
	Total	Small^a^	+SP^b^	+GPI-anchor^c^	+TMH^d^	
1A	11947	6743	793	55	1	295
1B	14047	8144	864	70	0	322
1D	11653	6384	808	66	1	285
2A	15186	8172	1176	81	0	339
2B	17266	9567	1268	86	0	392
2D	14982	7920	1235	83	0	361
3A	14391	7909	1103	81	1	352
3B	16845	9322	1227	85	0	419
3D	13769	7129	1109	71	0	342
4A	13791	7664	860	75	0	307
4B	11409	6403	754	59	2	270
4D	9600	5091	626	53	0	189
5A	14389	8094	949	72	1	319
5B	15355	8509	989	59	1	352
5D	13814	7360	981	73	0	307
6A	11533	6510	731	37	0	275
6B	14248	8207	847	36	0	307
6D	10383	5554	703	29	0	221
7A	15146	8608	1043	77	1	355
7B	14760	8442	914	70	0	305
7D	14430	7847	1009	68	1	314
Un	9830	6507	774	74	3	370

*^*a*^Small (≤250 amino acids) proteins. ^*b*^The number proteins with a signal peptide (predicted to be secreted). ^*c*^Small proteins, with a signal peptide and a predicted glycosylphosphatidylinositol anchor. ^*d*^Small proteins, with a signal peptide and predicted transmembrane helices (≥1). ^*e*^Predicted small, secreted proteins (with no GPI-anchor or TMH).*

The MEME-suite was used to identify sequence motifs in the signal peptide sequences of the proteins, using the cut-off of 10 motifs. Ten motifs were discovered in the sequence data with an *E*-value < 1e^–^^15^. When the motif sequences were realigned back to the sequences, we profiled which signal peptide sequences contained which motif. Each motif represented an average of 41 signal peptide sequences, suggesting that there is a lot of sequence dissimilarity and there are no motifs or common features found between all, or most, of the sequences. We clustered sequences based on their sequence similarity, clustering signal peptides that were >90% similar to each other. Only clusters with more than 3 sequences were considered true clusters, as signal peptides from homoeologous proteins almost exclusively clustered together, contributing to a high number of clusters with 2 or 3 proteins. By comparing the motifs within the clusters, we identified some clusters of signal peptides that all contained the same motif, but most of the clusters were not represented by any of the 10 motifs found. Due to the large number of small clusters, it seems that the SP sequences are generally too different from each other to analyze, and each cluster is represented by a different sequence motif. Therefore, we conclude that there are no defining motif features within wheat SSPs.

### *TaSSP* Gene Expression Was Induced During *Z. tritici* Infection

We mined microarray data to identify *TaSSPs* that were responsive to *Z. tritici* (isolate IPO323) at 4, 8, and 12 days post-inoculation in cultivars that were STB-resistant (cv. Stigg) or susceptible (cv. Gallant). From the microarray data, 5,163 genes in total, corresponding to 2,968 unique *Z. tritici* -responsive genes (some genes were differentially expressed at multiple timepoints or in both cultivars) were identified via the BLASTn algorithm corresponding Affymetrix probes to IWGSC refseq V1.1 genes IDs, and the proteins corresponding to these genes were retrieved from the protein annotation. In total, 198 SSPs were differentially expressed in the microarray study across the two cultivars and three timepoints (Table 2). These 198 genes/proteins correspond to 141 unique proteins, representing 4.7% of all differentially expressed genes/proteins. Of the 141 unique SSPs, 35 were uniquely differentially expressed in cv. Stigg, and 75 were uniquely differentially expressed in cv. Gallant. The remaining 27 SSPs were differentially expressed in both cultivars. The number of SSPs in the differentially expressed genes is significantly higher (χ^2^ *P*-value < 0.05) than the total percentage of SSPs across the wheat proteome (2.3%), indicating enrichment of SSPs in the disease response of wheat. Across the two cultivars, Stigg (STB-resistant) and Gallant (STB-susceptible), there was little difference in the percentage of differentially expressed genes that encoded SSPs, although a slightly higher percentage of SSPs was detected in Gallant (5.3%) as compared to Stigg (4.7%). Of the cv. Gallant-specific SSPs (75 SSPs), the most abundant biological processes and molecular functions were lipid transport and binding, cell surface receptor signaling, redox homeostasis, electron transfer, and peptidase activity. From the cv. Stigg-specific SSPs, the most dominant biological processes and molecular functions were the negative regulation of endopeptidase activity, lipid transport, and metal ion binding. A higher percentage of the differentially expressed genes were up-regulated (6.5%) versus down-regulated (3.5%) across both cultivars. The most striking difference was the temporal difference in SSP expression: 7.2% of the differentially expressed genes (DEGs) at 4 dpi across both cultivars were SSPs, compared to 4.6% at 8 dpi and 3.3% at 12 dpi (Table 2). From the *Z. tritici*-responsive SSPs, we chose two for further characterization based on their fold change in cv. Stigg compared to cv. Gallant; TaSSP6 and TaSSP7 (Table 3). *TaSSP6* was represented by the microarray probe Ta.23397.1.S1_x_at, which was upregulated in cv. Stigg at 4 dpi by 4.1-fold, but was not differentially expressed in cv. Gallant at 4 dpi. This probe was upregulated in cv. Gallant at 8 dpi by 6.3-fold, but was not differentially expressed in cv. Stigg at 8 dpi. *TaSSP7* was represented by the probe Ta.28289.2.S1_x_at, which was upregulated at 8 dpi in cv. Stigg by 1.7-fold, and at 12 dpi in cv. Stigg by 44.7-fold. *TaSSP7* was not differentially expressed in cv. Gallant. Therefore, *TaSSP6* is *Z. tritici* -responsive in both cvs. Stigg and Gallant, but was upregulated earlier in Stigg than Gallant, and *TaSSP7* is specific to cv. Stigg (at least at the time points explored). *TaSSP6* consists of three homoeologues on the group 2 chromosomes; the A homoeologue encodes one splice variant, the B homoeologue encodes three splice variants, and the D homoeologue encodes two splice variants. All six variants of *TaSSP6* encode for small, secreted proteins. BLASTx of the *TaSSP6* homoeologues and variants revealed that 6 of the *TaSSP6* variants had significant homology to a glycine rich protein, and one (*TaSSP6-D.1*), had homology to a probable H/ACA ribonucleoprotein complex subunit 1 (Table 4). Four of the *TaSSP6* variants had a hit to the PANTHER classification system^4^ domain PTHR37389, which is an uncharacterized protein domain in wheat. However, in *Z. mays* and *Nicotiana tabacum* this domain ID is described as a glycine-rich protein domain. The only gene ontology (GO) terms associated with these genes were the biological process cell wall organization and the cellular components extracellular region and cell wall. *TaSSP7* consists of three homoeologues on the group 3 chromosomes, each with one splice variant. All three *TaSSP7* genes encode small, secreted proteins. *TaSSP7-A.1* had significant homology to a papilin-like isoform, while *TaSSP7-B.2* had no BLASTx description, and *TaSSP7-D.1* had homology to a Kunitz/Bovine pancreatic trypsin inhibitor domain protein (Table 4). None of the *TaSSP7* homoeologues had any domains, based on the InterProScan. The GO terms associated with the A and D-genome homoeologues were the biological processes chitin metabolic process, proteolysis, and negative regulation of peptidase activity, the molecular functions serine-type endopeptidase inhibitor activity, chitin binding, and peptidase activity, and the cellular component associated with these genes was the extracellular region. No GO terms were associated with the B-genome homoeologue. Although multiple GO terms were associated with TaSSP7, no protein domains were found within these sequences from the InterProScan search.

**TABLE 2:** The number of STB-responsive SSPs from a microarray of wheat cultivars Gallant (susceptible) and Stigg (resistant) at 4, 8, and 12 days post inoculation with *Zymoseptoria tritici^a^*.

Cultivar	Regulation	Timepoint (DPI)	Number of proteins			GPI-Anchor^e^	Small and secreted^f^ (%)
			
			DEGs^b^	Small^c^	+Signal peptide^d^		
Gallant	Up	4	33	6	15	0	4(12.1)
		8	1063	312	121	2	32(3)
		12	1321	366	250	13	63(4.7)
	Down	4	51	11	13	2	3(5.8)
		8	204	64	21	2	9(4.4)
		12	600	164	62	3	14(2.3)
Stigg	Up	4	37	12	5	0	4(10.8)
		8	273	66	66	1	15(5.5)
		12	1174	342	127	2	38(3.2)
	Down	4	4	0	0	0	0(0)
		8	122	38	17	2	7(5.7)
		12	281	75	38	0	9(3.2)

*^*a*^Microarray probes were converted to genes using BLAST, and signal peptides were predicted with SignalP V5.0 standalone. ^*b*^DEGs, Differentially expressed genes that corresponded to proteins. ^*c*^Small (≤250 amino acids) proteins responsive to Z. tritici. ^*d*^The number of the differentially expressed genes that code for a protein with a signal peptide (predicted to be secreted). ^*e*^Small, differentially expressed proteins, with a signal peptide and a predicted glycosylphosphatidylinositol anchor. ^*f*^The number and percentage of differentially expressed proteins that are predicted to be small, secreted proteins.*

**TABLE 3:** Characteristics of selected *Z. tritici* – responsive small, secreted proteins.

							Cleavage site^e^	
							
Affymetrix probe/	Days post	Fold change in	% Identity/	IWGSC	Length			
Gene ID^a^	inoculation^b^	cv. Stigg	*E*-value^c^	ID	(AA)	P (SP)^d^	Position	Sequence
Ta.23397.1.S1_x_at (*TaSSP6*)	4	4.11	92.9/3.6E^–^^92^	TraesCS2A02G513900.1	164	0.99	27–28	TQA-KK
			94/3.1E^–^^80^	TraesCS2B02G542000.1	120	0.99	27–28	TQA-KK
			94/3.1E^–^^80^	TraesCS2B02G542000.2	118	0.99	27–28	TQA-KK
			94/3.1E^–^^80^	TraesCS2B02G542000.3	152	0.99	27–28	TQA-KK
			98.3/3.1E^–^^117^	TraesCS2D02G515500.1	152	0.99	27–28	TQA-KK
			98.3/3.1E^–^^117^	TraesCS2D02G515500.2	126	0.99	27–28	TQA-KK
Ta.28289.2.S1_x_at (*TaSSP7*)	12	44.7	92.3/2.9E^–^^11^	TraesCS3A02G095900.1	141	0.91	27–28	MMA-VT
			89.4/3.2E^–^^17^	TraesCS3B02G111700.1	115	0.99	28–29	ADA-SA
			95.1/7.2E^–^^9^	TraesCS3D02G096300.1	138	0.98	28–29	TDA-SA

*^*a*^ID of the Affymetrix probe from the wheat 61K Affymetrix microarray. These probes were differentially expressed by Z. tritici treatment and encoded small, secreted proteins. ^*b*^Days post inoculation (with Z. tritici). ^*c*^% identity and E-value of the Affymetrix probe sequence to the IWGSC V1.1 reference gene annotation. ^*d*^Likelihood probability of a signal peptide (based on SignalP v5.0). ^*e*^The cleavage site is the site of cleavage of the signal peptide from the nascent protein.*

**TABLE 4:** BLASTx hits and InterProScan domain IDs for the *TaSSP* genes.

Gene	BLASTx description	*E*-value of BLASTx hit	InterPro ID
*TaSSP6-A.1*	Glycine rich protein	8.20E^–^^17^	–
*TaSSP6-B.1*	Glycine-rich cell wall structural protein precursor	3.60E^–^^17^	PTHR37389
*TaSSP6-B.2*	Glycine-rich cell wall structural protein precursor	3.43E^–^^17^	PTHR37389
*TaSSP6-B.3*	Glycine rich protein	3.16E^–^^17^	–
*TaSSP6-D.1*	Probable H/ACA ribonucleoprotein complex subunit 1	2.43E^–^^72^	PTHR37389
*TaSSP6-D.2*	Glycine-rich cell wall structural protein precursor	2.19E^–^^22^	PTHR37389
*TaSSP7-A.1*	Papilin-like isoform X2	6.09E^–^^87^	–
*TaSSP7-B.1*	Unnamed protein product	5.55E^–^^75^	–
*TaSSP7-D.1*	Kunitz/Bovine pancreatic trypsin inhibitor domain protein	7.10E^–^^79^	–

Both *TaSSP6* and *TaSSP7* were cloned from cv. Stigg. *TaSSP6-2B* from cv. Stigg has 97% identity to the Chinese Spring sequence, with 5 single nucleotide polymorphisms (SNPs) within the gene sequence between *TaSSP6-2B* from cv. Stigg and *TaSSP6-2B* from cv. Chinese Spring. *TaSSP6-2B* has 94.6% identity to *TaSSP6-2D* and 93.7% identity to *SSP6-2A*. *TaSSP7-3A* was cloned from cv. Stigg and has 97.5% identity to the cv. Chinese Spring reference sequence, with 7 SNPs in *TaSSP7-3A* between the two cultivars. *TaSSP7-3A* has 94.6% identity to cv. Chinese spring *TaSSP7-3D*, and 84.3% identity to *TaSSP7-3B.* qRT- PCR primers for each gene were designed to amplify all homoeologues of both *TaSSP* genes. These were used to assess the expression of *TaSSP6* and *TaSSP7* in response to another isolate of *Z. tritici* (Cork Cordiale 4) in wheat seedlings of cvs. Stigg and Gallant at 4, 8, and 12 dpi. qRT-PCR of *Ta*SSP6 revealed an increase in expression in the *Z. tritici*-treated samples at 8 dpi. While this difference was significant in a *t*-test (*P* < 0.05), it was not significant once corrected for multiple comparisons (Tukey’s *post hoc* test). qRT-PCR of *TaSSP7* showed an increase in transcript abundance in *Z. tritici*-treated plants at 12 dpi, but this difference was not significant. Expression of both *Ta*SSP6 and *TaSSP7* was much greater in cv. Stigg than in cv. Gallant in both treated and control conditions (Supplementary Figure S3).

### *TaSSPs* Have Functional Secretion Signals

**FIGURE 3:**
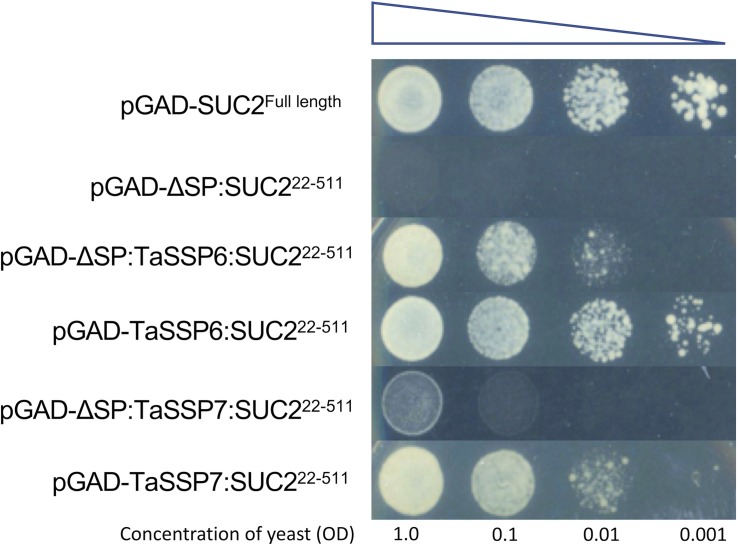
Validating the cellular secretion of wheat small secreted proteins (TaSSPs) secretion using a yeast expression system. The expression of the *TaSSPs* with a positive secretion signal will result in the secretion of the SUC2 protein allowing the growth of yeast on sucrose-containing media. The TaSPP6 and TaSSP7 proteins with secretion signal removed cannot grow on media. The yeast strain transformed with SUC2^*FullLength*^ (with signal peptide) gene was used as a positive control while the strain transformed ΔSP:SUC2^22^^–^^511^ (without signal peptide) gene was used as a negative control. Yeast was spotted onto the media in a serial dilution from an initial OD_600_ of 1.0 to 0.1, 0.01, and 0.001, respectively.

To test the secretion of wheat TaSSPs, a complementation assay system in which the survival of the host depends on the secretion of the protein of interest was chosen. The sucrose transport protein (*SUC2*) gene of *S. cerevisiae* strain SEY6210 was completely knocked out to generate a *suc2* mutant yeast strain. Because the *suc2* gene of the yeast was replaced by tryptophan synthesis (*Trp1*) gene by homologous recombination, the *suc2* mutant yeast can grow on Trp dropout (-Trp) yeast media plate with glucose as a carbon supply, but it cannot grow on media containing sucrose as the sole energy source without the presence of a secreted SUC2 protein. A series of expression vectors were developed for the secretion assay in yeast (Supplementary Figure S2): the pGADT7 vector was used as a backbone expression vector containing a yeast alcohol dehydrogenase promotor (pADH1) to drive gene expression. The pGAD-SUC2^*F*^^*ulllength*^ was used as a positive control, and the pGAD-ΔSP:SUC2^22^^–^^511^ (without signal peptide) was used as a negative control. The Gateway-compatible pGAD-GW-SUC2^22^^–^^511^ vector was used for TaSSPs protein yeast secretion assay, wherein a linker (HA tag-Kex2 cleavage site) was added between Gateway Reading Frame Cassette and truncated *SUC2* gene. The Kex2 cleavage site improved yeast secretion productivity and ensured fusion proteins did not affect the SUC2 activity. The *TaSSPs* genes were fused to the N-terminus of SUC^22^^–^^511^ gene, and the recombinant genes were then expressed in the *suc2* mutant yeast strains (as were the positive and negative expression vectors pGAD-SUC2^*F*^^*ulllength*^ and pGAD-ΔSP:SUC2^22^^–^^511^). The result showed that all *suc2* mutant yeast cells containing pGAD-TaSSPs:SUC2^22^^–^^511^ (full length TaSSPs, including their signal peptide) vector grew on Trp and Leu dropout (-TL) plates, using sucrose as the sole carbon source. When the signal peptide of TaSSP6 and TaSSP7 were deleted, the associated yeast cells either failed to grow (pGAD-ΔSP:TaSSP7:SUC2^22^^–^^511^) or growth was diminished (pGAD-ΔSP:TaSSP6:SUC2^22^^–^^511^). In conclusion, the TaSSPs have a functional secretion signal peptide that can complete the function of the signal peptide of SUC2 protein in yeast. Thus we concluded that these TaSSP proteins are secreted (Figure 3).

### Silencing *TaSSPs* Enhances Wheat Susceptibility to *Z. tritici*

Virus induced gene silencing (VIGS) was used to study the function of *TaSSP6* and *TaSSP7*, to determine if reducing transcript levels of *TaSSP6* and *TaSSP7* altered the phenotypic response to *Z. tritici* isolate Cork Cordiale 4 in the STB-resistant cv. Stigg. Two different VIGS fragments were designed to silence all homoeologues of each *TaSSP* gene.

**FIGURE 4:**
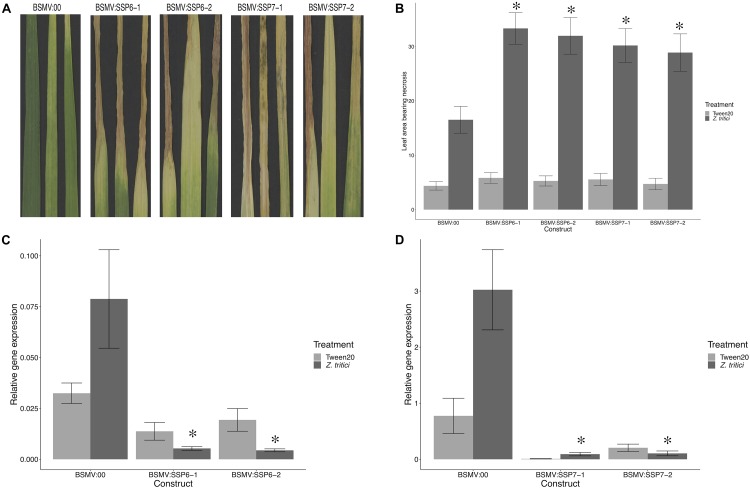
Virus-induced gene silencing (VIGS) of *TaSSPs* increases susceptibility to STB disease in the STB-resistant cv. Stigg. **(A)** Representative photos of STB-treated leaves of plants that were inoculated with either the empty vector (BSMV:00) or a silencing vector. **(B)** Leaf area showing necrosis was significantly higher (^∗^) in plants with silenced *TaSSP6* and *TaSSP7* that were treated with *Zymoseptoria tritici*. Both silencing constructs of both *TaSSP* genes had the same effect on the disease response. Silencing of *TaSSP6* **(C)** and *TaSSP7* **(D)** was confirmed via qRT-PCR; both constructs of both *TaSSP* genes significantly (^∗^*P* < 0.05) reduced transcript abundance of the genes in plants treated with *Z. tritici*.

The phenotype of silenced plants was assessed by scoring the percentage of leaf area bearing necrosis at 21 dpi. Pycnidia coverage on the leaves after 10 days in 100% humidity confirmed that the STB disease developed as expected on the *Z. tritici*-treated leaves, and that no STB disease developed on the control leaves (Supplementary Figure S4). VIGS silencing of *TaSSP6* caused a significant (*P* < 0.01) increase in necrosis by 2-fold (construct BSMV:TaSSP6-V1) and 1.9-fold (construct BSMV:TaSSP6-V2). Silencing of *TaSSP7* caused a significant (*P*-value < 0.05) increase in necrosis by 1.8-fold (construct BSMV:TaSSP7-V1) and 1.7-fold (construct BSMV:TaSSP7-V2). There were no significant differences in disease levels between the two constructs for each gene (Figures 4A,B). The efficiency of *TaSSP* silencing were confirmed by qRT-PCR. *TaSSP6* and *TaSSP7* expression was induced by *Z. tritici* in the BSMV:00 plants at the timepoint analyzed, but this difference was not significant. In plants treated with *Z. tritici*, VIGS silencing of *TaSSP6* caused a significant (*P* < 0.01) decrease in transcript abundance of *TaSSP6* by 18-fold (construct BSMV:TaSSP6-V1) and 22-fold (construct BSMV:TaSSP6-V2) (Figure 4C). Silencing of *TaSSP7* caused a significant (*P*-value < 0.05) decrease in transcript abundance of *TaSSP7* by 24-fold (construct BSMV:TaSSP7-V1) and 23-fold (construct BSMV:TaSSP7-V2) (Figure 4D). VIGS silencing of both *TaSSP* genes did not significantly reduce gene expression in the control plants (treated with 0.02% Tween20).

### TaSSPs Interact With Fungal Small, Secreted Proteins

**FIGURE 5:**
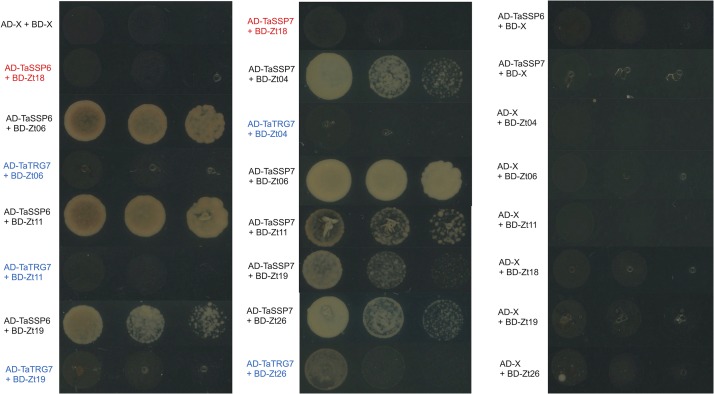
TaSSP proteins interact with ZtSSPs in yeast. Matchmaker Gold yeast strains carrying the bait vector pGADT7 containing TaSSPs were transformed with the prey vector pGBKT7 containing ZtSSPs. Strains were spotted on synthetic defined (SD) selective media (lacking leucine, tryptophan, histidine, and adenine, -LTHA) and incubated at 30°C for 3 days. Wheat protein TaTRG7 (in blue) was used as a negative control for the TaSSPs and Zt18 was used as a negative control for *Z. tritici* SSPs (in red). Empty vector controls are shown in the right-hand panel.

**FIGURE 6:**
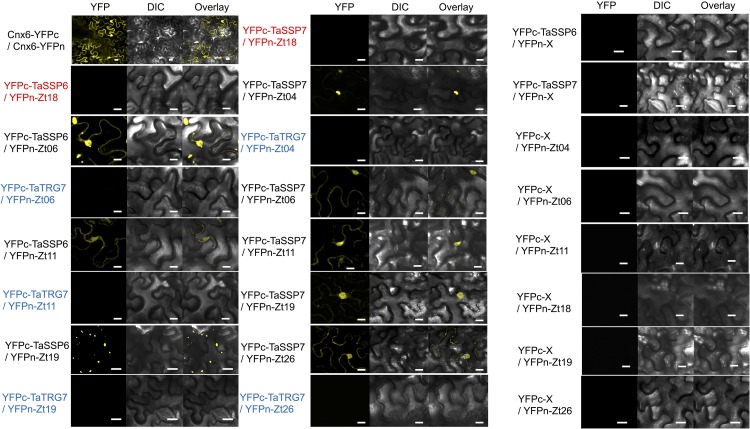
Bimolecular fluorescence complementation analysis of TaSSPs and ZtSSPs interactions in *Nicotiana benthamiana*. Cnx6 homodimerization *in planta* was used as a BiFC positive control. Wheat protein TaTRG7 (in blue) was used as a negative control for the TaSSPs and Zt18 was used as a negative control for *Z. tritici* SSPs (in red). Bars = 10 μm. Empty vector controls are shown in the right-hand panel.

We hypothesized that one or more of TaSSP6 and TaSSP7 may interact with ZtSSPs. We used yeast two-hybrid (Y2H) analysis to identify ZtSSPs that can physically interact with TaSSP proteins. Using TaSSP6 or TaSSP7 as bait, we screened the interaction with 27 ZtSSPs (Supplementary Table S2) using a galactose-responsive transcription factor GAL4 (GAL4)-based yeast two-hybrid system. The results showed that TaSSP6 could interact with three ZtSSPs, and TaSSP7 could interact with five ZtSSPs in yeast. Three of the ZtSSPs were common to TaSSP6 and TaSSP7 (Figure 5). Zt18 was used as a negative control for ZtSSPs, and the wheat STB-responsive non-secreted protein TaTRG7 protein was used as a negative control for the TaSSP proteins, as it was previously demonstrated not to interact with these ZtSSPs (Brennan et al., 2020, in press). Based on Y2H results, we deduced that TaSSP6 interacted with three separate ZtSSPs: Zt06, Zt11 and Zt19. TaSSP7 interacted with five ZtSSPs: Zt04, Zt06, Zt11, Zt19, and Zt26. We then investigated whether the TaSSPs and ZtSSPs could interact *in planta* using BiFC assays. The N-terminal part of YFP was fused to the N-terminal of TaSSPs (without the signal peptide) to create YFPn-TaSSP6 and YFPn-TaSSP7. The C-terminal part of YFP was fused to the N-terminal of the ZtSSPs (without the signal peptide) to create YFPc-Zt04, YFPc-Zt06, YFPc-Zt11, YFPc-Zt19, and YFPc-Zt26 fusion. YFPn-TaTRG7 and YFPc-Zt18 were used as negative controls. Using *Agrobacterium*-mediated transient co-expression in *N. benthamiana*, interactions between these fusion proteins were assayed for YFP fluorescence using a Confocal Laser Scanning Microscope at 2 days post-infiltration. We observed a strong YFP signal in the cytoplasm of leaf cells co-infiltrated with *A. tumefaciens*. Based on fluorescent signals we deduced that TaSSP6 interacted with ZtSSPs Zt06, Zt11, and Zt19, and TaSSP7 interacted with Zt04, Zt06, Zt11, Zt19, and Zt26 (Figure 6). Thus, BiFC confirmed that the interactions occurring in yeast also occurred *in planta.* We used a BLASTx search to the NCBI non-redundant protein database and InterProScan analysis to identify if any of the *ZtSSP* genes had any known domains or predicted function. None of Zt04, Zt06, Zt11, Zt19, or Zt26 had any known domains or predicted function from the BLASTx search, and none had any domains based on the InterProScan analysis.

### Fungal SSPs That Interact With TaSSPs Induce Cell Death in the Non-host *N. benthamiana*

**FIGURE 7:**
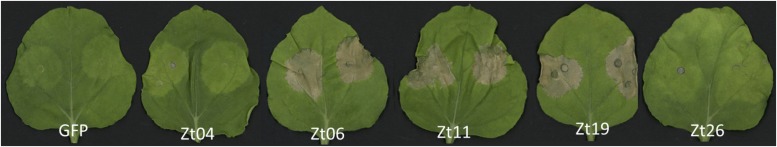
ZtSSPs induced cell death in *N. benthamiana* leaves. The candidate ZtSSPs, which interacted with TaSSPs, were expressed in leaves of *N. benthamiana* by *Agrobacterium*-mediated expression. Three of the ZtSSPs induced cell death phenotypes. GFP was expressed as negative control. Leaves photographed at 7 days post infiltration (dpi).

We tested the ability of ZtSSPs that interacted with TaSSPs to induce cell death in tobacco leaves. A high-level and long-lasting protein expression vector pEAQ-HT (Sainsbury et al., 2009) was used to express the six ZtSSPs via an *Agrobacterium*-mediated transient expression assays in *N. benthamiana* leaves. This system had been successfully used for ZtSSP expression in *N. benthamiana* (Kettles et al., 2017). The results showed that three of the ZtSSPs (Zt06, Zt11, and Zt19, all of which interact with both TaSSP6 and TaSSP7) induced cell death in *N. benthamiana* leaves (Figure 7). The other two ZtSSPs, Zt04 and Zt26, which interact with TaSSP7, did not induce cell death. The GFP alone was also transiently expressed as a negative control and no cell death was detected in GFP-expressing control leaves. These data thus showed that a subset of ZtSSPs that interact with TaSSP proteins can induce cell death in the non-host plant *N. benthamiana*. To test whether the interaction between TaSPPs and ZtSSPs affected cell death, the interacted TaSPP and ZtSSP combinations were co-expressed in *N. benthamiana* leaves, and similar phenotypes could be observed (Supplementary Figure S5), suggesting that the TaSSP-ZtSSP interaction did not affect the cell death phenotype *in planta*, at least in *N. benthamiana*. The TaSSP proteins did not induce a cell death phenotype when infiltrated into *N. benthamiana* leaves alone (Supplementary Figure S5).

## Discussion

The apoplastic space is one of the first sites of conflict between plant and pathogen. In the case of STB disease of wheat, the fungus is purely apoplastic, and the conflict between fungal effectors and plant proteins determines the outcome of the disease progression (Doehlemann and Hemetsberger, 2013). In this study, we identified numerous wheat small secreted proteins (SSPs) expressed during the interaction with the fungal pathogen *Z. tritici* and showed that SSPs can enhance wheat resistance to STB disease.

Until recently, large-scale or automated identification or characterization of gene families in wheat was difficult due to the lack of an annotated reference genome. However, since the release of the IWGSC refseq (IWGSC, 2018), we were able to automate the discovery of predicted SSPs from the protein annotation by writing a wrapper script to survey protein length and predict the presence of a signal peptide using SignalP v5.0 (Armenteros et al., 2019). By using this pipeline, we discovered that 58% of all wheat proteins were smaller than 250 amino acids in length, and a positive-skew in the distribution of protein length revealed that smaller proteins were more abundant in the genome than longer proteins. Plant-specific proteins are known to be on-average shorter than those of animals and fungi as they generally contain fewer exons than the genomes of the other eukaryotic kingdoms (Ramirez-Sanchez et al., 2016). Due to their lower capacity to house domains, and limited folding potential, small (<200 AA) proteins are usually limited in function (Chothia et al., 2003), but are known to be important for multiple biological processes, including the stress response (Storz et al., 2014).

We used SignalP to predict the presence of signal peptide sequences at the N terminal of the wheat proteins. Signal peptides are found on secreted as well as transmembrane proteins, and also in proteins within cellular organelles (Armenteros et al., 2019). Signal peptides were predicted in 7% of the wheat proteins, and all of these proteins were predicted to be secreted via the general secretory pathway; protein translocation across the endoplasmic reticulum membrane (Vitale and Denecke, 1999). Combining the secretion predictions with protein length, and filtering out proteins with any transmembrane domains and GPI anchors, we identified 6,998 proteins that were small, and had a secretion signal (SSPs). We used Blast2Go to functionally annotate the *TaSSP* genes and test for any enrichment of function. Many gene ontology terms were significantly enriched in the *TaSSP* gene set, including many GO terms that are important for the disease response. These included pathways and cellular components that have been characterized and implicated in the plant defense response, including peptidase inhibitor activity (Benbow et al., 2019), and the apoplastic space (Doehlemann and Hemetsberger, 2013; Jashni et al., 2015).

Using the *Z. tritici* microarray data set from Brennan et al. (2020), we identified *Z. tritici*-responsive SSPs. We found a significant enrichment of SSPs in the STB-response of wheat, indicating that SSPs are over-represented in the wheat disease response to *Z. tritici*. Additionally, we observed a temporal decrease in the number of SSPs that were differentially expressed in response to *Z. tritici*; from 8% at 4 dpi to 3.7% at 12 dpi. Four dpi is well within the latent phase of the disease, and is at the tail end of a peak in expression of *Z. tritici* effectors associated with the latent phase of the disease (Mirzadi Gohari et al., 2015). It seems that expression of the *TaSSP* genes studied here may be related to that of *ZtSSP*s. We hypothesize that many of these *TaSSP* genes evolved in response to pathogen attack, and as a mechanism for effector-triggered immunity in the wheat-*Z. tritici* pathosystem.

Of the *Z. tritici*-responsive SSPs, we chose to focus on two, *TaSSP6* and *TaSSP7*, because they had a high fold change in the STB-resistant cv. Stigg, and were not differentially expressed in the susceptible cv. Gallant in response to isolate IPO323. The probes that were differentially expressed from the Brennan et al. (2020, in press) microarray study were used to identify genes from the IWGSC refseq v1.1 annotation, and we found that both genes were present as 3 homoeologues, and *TaSSP6* could be alternatively spliced into five isoforms (1 × A, 3 × B, 2 × D). TaSSP6 is a putative glycine-rich protein, and four out of the six *TaSSP6* variants had hits to the domain PTHR37389 – an uncharacterized domain in wheat. The PTHR37389 domain has 18 subfamilies, including glycine-rich protein-like and cold and drought regulated protein-like, suggesting that TaSSP7 contains a variant or subfamily of PTHR37389 that may be associated with the stress response. The GO terms associated with TaSSP6 were cell wall organization, the extracellular region, and the cell wall. The cell wall, and genes involved in cell wall reorganizing/cell wall remodeling have previously been associated with wheat resistance to STB, with increased activity of cell wall remodeling and reinforcement in a resistant wheat cultivar in response to *Z.* tritici (Yang et al., 2015). *TaSSP7-A.1* has homology to a papilin-like isoform, *TaSSP7-B.1* to an unnamed protein product, and *TaSSP7-D.1* to a trypsin protease inhibitor domain protein, although no domains were found any of the three homoeologues. Both papilin-like proteins and trypsin protease inhibitors are serine-type endopeptidase inhibitors (serpins), that are known to play a role in the disease response in wheat and other plant species (Bhattacharjee et al., 2017; Bao et al., 2018; Benbow et al., 2019). However, none of the TaSSP7 homoeologues were characterized as wheat serpins in a recent genome wide characterization of the wheat serpin family (Benbow et al., 2019), indicating that although TaSSP7 may have partial homology to serpin-like proteins, it doesn’t contain any of the serpin protein domains.

As the microarray study provides no information on homoeologue specificity, we cannot be sure which homoeologue (and indeed isoform) is contributing to the differential expression of the probe. The independent time course generated for gene expression studies of the *TaSSP* genes used a different isolate of *Z. tritici*: the aggressive Irish field isolate Cork Cordiale 4 was used rather than the reference isolate IPO323. While we saw an increase in expression of *TaSSP6* in cv. Stigg at 8 dpi, this difference was not significant, partly due to the large variance in gene expression in the leaves. Additionally, we saw no significant difference in *TaSSP7* expression in our time course. We attribute this, in part, to a change in *Z. tritici* isolate used for the expression study.

In addition to their expression in response to *Z. tritici*, TaSSP6 and TaSSP7 were predicted to be secreted proteins, based on the presence of a signal peptide sequence at the N-terminus of the protein. To validate this prediction, a complementation-based secretion assay was used based on that of Plett et al. (2017). This system confirmed that both TaSSP6 and TaSSP7 have functional secretion signal peptides and are secreted. TaSSP7 that lacked its signal peptide was not secreted, indicating that the signal peptide is vital for secretion of TaSSP7. However, TaSSP6 was secreted (although to a lesser extent) once the signal peptide was removed. This phenomenon, known as leaderless secretion, is known to occur in bacteria (Bendtsen et al., 2005), and has been characterized in plants, where a normally non-secreted, cytoplasmic protein was secreted into the plant apoplast in response to SA signaling (Cheng et al., 2009). While TaSSP6 is ordinarily secreted and has a functional signal peptide, its secretion (at least in yeast) was improved by, but not wholly dependent on its signal peptide. Further study is warranted here to determine if TaSSP6 is secreted *in planta* without its signal peptide.

To test the function of *TaSSP6* and *TaSSP7*, both genes were transiently silenced using the VIGS system. The expression of both *TaSSP* genes was induced by *Z. tritici* in the BSMV:00 (empty vector) plants, but the difference in gene expression between *Z. tritici* and Tween20 treated plants was not significant. This is likely due to the use of a different *Z. tritici* in both the temporal gene expression study and the VIGS, where we used the isolate Cork Cordiale 4 versus IPO323 that was used in the original microarray study. In plants silenced with either *TaSSP* gene, the expression of the *TaSSP* gene was significantly reduced by the silencing construct, and a significant increase in STB disease was observed, demonstrating a ∼2-fold increase in susceptibility of the STB-resistant cv. Stigg. It seems both genes, when silenced, give a similar phenotype and may serve to shorten the latent phase of cv. Stigg. Normally ∼35 days long, Stigg’s lengthy latent phase contributes to its exceptional STB resistance in the field (Hehir et al., 2018). With a somewhat elusive pedigree, Stigg’s various chromosomal introgressions from wild wheat relatives are thought to contribute to its resistance, and the key players behind this characteristic are largely unknown. We suggest that, based on their expression and function, *TaSSP6* and *TaSSP7* are involved in the latent phase of infection and stave off disease by interacting with fungal small, secreted proteins. Supporting this is the confirmation that both TaSSP6 and TaSSP7 interact with ZtSSPs *in vitro* and *in planta*. Both TaSSPs interact with three common ZtSSPs: Zt06, Zt11, and Zt19. Interestingly, it was these three ZtSSPs that could also induce cell death in tobacco leaves. Although tobacco is a non-host to *Z. tritici*, these proteins were clearly recognized and responded to, suggesting the potential for activation of down-stream signaling cascades that can promote a hypersensitive response. However, co-expression of the TaSSPs with the ZtSSPs into the tobacco leaves did not alter the cell death phenotype, suggesting that the cell death phenotype depends on non-host resistance (Kettles et al., 2017). These ZtSSPs did not have any known domains, based on InterProScan, so we cannot hypothesize as to their function or method of interaction with TaSSPs, but as infection of wheat cv. Stigg with *Z. tritici* does not elicit a hypersensitive response, we must conclude that the elicitation of a host response by fungal SSPs is interfered with by host defense genes. In this case, we propose a role for *TaSSP6* and *TaSSP7* to stop, delay, or alter the effect of the ZtSSPs on the cytology of the host.

In summary, we present two novel wheat genes that encode for small, secreted proteins, and contribute to resistance to STB disease. The wheat proteins interact with fungal small, secreted proteins and this interaction may contribute the resistance phenotype observed in cv. Stigg. We hypothesize that these TaSSP proteins may be important for effector-triggered immunity in wheat infected with STB disease. This study gives insight into the complex mechanisms of the host-pathogen interaction in this economically important disease, and further characterization of wheat small, secreted proteins, especially those that are responsive to disease, may reveal insights into the evolution of effector-triggered immunity in plants.

## Data Availability Statement

The microarray data from Brennan et al. (2020) is available at 10.6084/m9.figshare.11882601.v1. The raw data supporting the conclusions of this article will be made available by the authors, without undue reservation, to any qualified researcher.

## Author Contributions

BZ, HB, and FD designed the experiments. BZ, HB, CB, and CA carried out all experiments. BZ and HB analyzed the data. SK and AF identified SSPs from *Z. tritici*. BZ and HB wrote the manuscript. EM, JB, and FD reviewed the manuscript.

## Conflict of Interest

The authors declare that the research was conducted in the absence of any commercial or financial relationships that could be construed as a potential conflict of interest.

## Footnotes

1 https://urgi.versailles.inra.fr/download/iwgsc/IWGSC_RefSeq_Annotations/v1.1/, accessed January 2020

2 http://www.affymetrix.com/Auth/analysis/downloads/data/wheat.probe_fasta.zip, accessed January 2020

3 http://plants.ensembl.org/Triticum_aestivum/Info/Index

4 http://www.pantherdb.org/
