# Supplementary material for: Wheat Encodes Small, Secreted Proteins That Contribute to Resistance to Septoria Tritici Blotch
Source: Front Genet. 2020 May 12;11:469. doi: 10.3389/fgene.2020.00469 (PMC7235427; doi:10.3389/fgene.2020.00469)
Supplement: Supplementary file 2 [file Data_Sheet_2.docx]

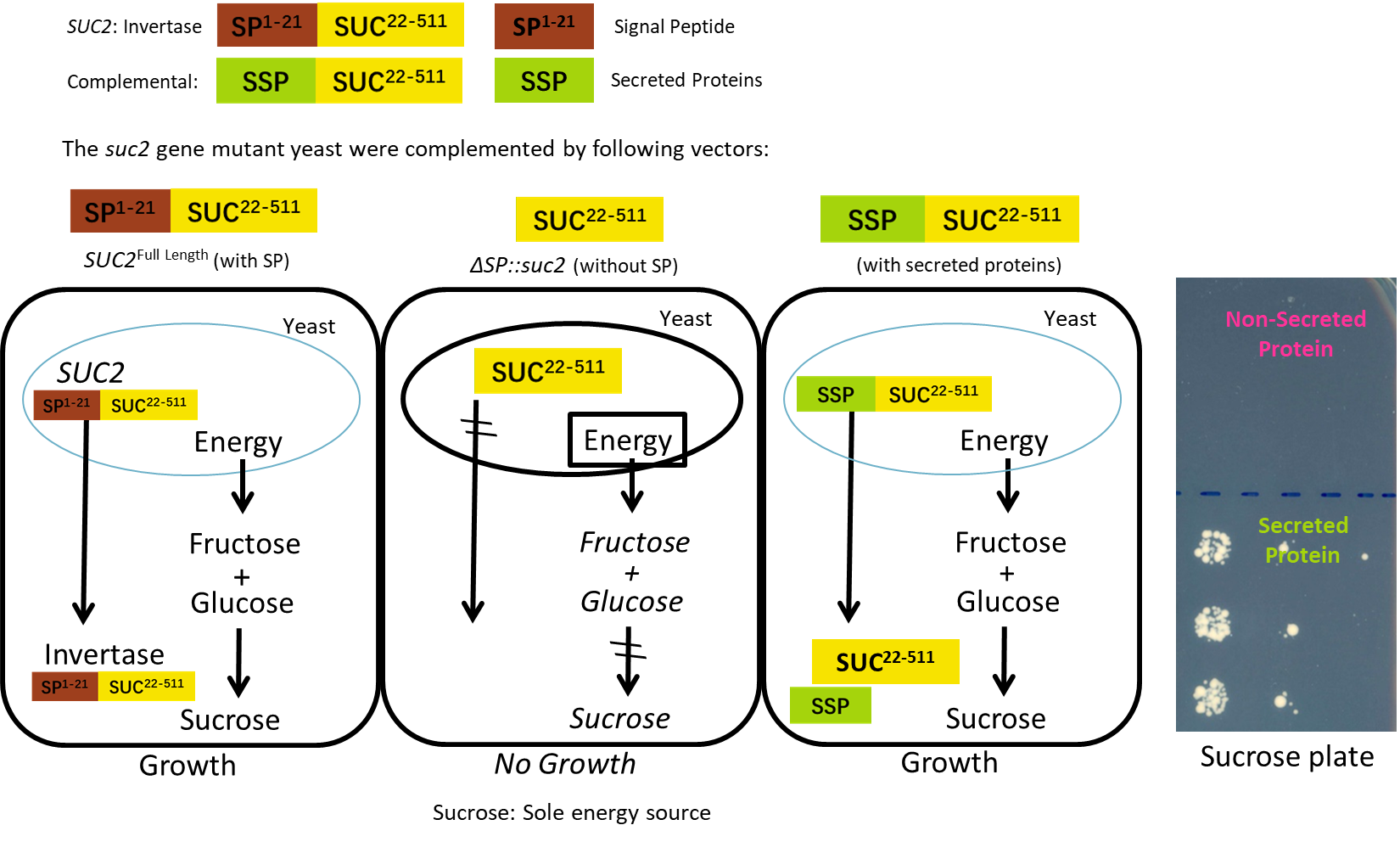


**Supplemental Figure S1** Schematic diagram of the yeast TaSSP secretion assay. *SUC2*: Sucrose transport protein, SP^1-21^: signal peptide of *SUC2*. SP^22-511^: truncated *SUC2* gene (without signal peptide) with hydrolase activity, SSP: Small Secreted Peptide.

**(A)**
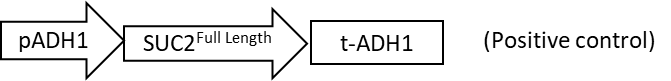


> ADH1 promoter-Full length SUC2 (with Signal peptide)-ADH1 terminator

ATCCTTTTGTTGTTTCCGGGTGTACAATATGGACTTCCTCTTTTCTGGCAACCAAACCCATACATCGGGATTCCTATAATACCTTCGTTGGTCTCCCTAACATGTAGGTGGCGGAGGGGAGATATACAATAGAACAGATACCAGACAAGACATAATGGGCTAAACAAGACTACACCAATTACACTGCCTCATTGATGGTGGTACATAACGAACTAATACTGTAGCCCTAGACTTGATAGCCATCATCATATCGAAGTTTCACTACCCTTTTTCCATTTGCCATCTATTGAAGTAATAATAGGCGCATGCAACTTCTTTTCTTTTTTTTTCTTTTCTCTCTCCCCCGTTGTTGTCTCACCATATCCGCAATGACAAAAAAATGATGGAAGACACTAAAGGAAAAAATTAACGACAAAGACAGCACCAACAGATGTCGTTGTTCCAGAGCTGATGAGGGGTATCTCGAAGCACACGAAACTTTTTCCTTCCTTCATTCACGCACACTACTCTCTAATGAGCAACGGTATACGGCCTTCCTTCCAGTTACTTGAATTTGAAATAAAAAAAAGTTTGCTGTCTTGCTATCAAGTATAAATAGACCTGCAATTATTAATCTTTTGTTTCCTCGTCATTGTTCTCGTTCCCTTTCTTCCTTGTTTCTTTTTCTGCACAATATTTCAAGCTATACCAAGCATACAATCAACTCCAGAATTCAGCTTTGCAAAGATGCTTTTGCAAGCTTTCCTTTTCCTTTTGGCTGGTTTTGCAGCCAAAATATCTGCATCAATGACAAACGAAACTAGCGATAGACCTTTGGTCCACTTCACACCCAACAAGGGCTGGATGAATGACCCAAATGGGTTGTGGTACGATGAAAAAGATGCCAAATGGCATCTGTACTTTCAATACAACCCAAATGACACCGTATGGGGTACGCCATTGTTTTGGGGCCATGCTACTTCCGATGATTTGACTAATTGGGAAGATCAACCCATTGCTATCGCTCCCAAGCGTAACGATTCAGGTGCTTTCTCTGGCTCCATGGTGGTTGATTACAACAACACGAGTGGGTTTTTCAATGATACTATTGATCCAAGACAAAGATGCGTTGCGATTTGGACTTATAACACTCCTGAAAGTGAAGAGCAATACATTAGCTATTCTCTTGATGGTGGTTACACTTTTACTGAATACCAAAAGAACCCTGTTTTAGCTGCCAACTCCACTCAATTCAGAGATCCAAAGGTGTTCTGGTATGAACCTTCTCAAAAATGGATTATGACGGCTGCCAAATCACAAGACTACAAAATTGAAATTTACTCCTCTGATGACTTGAAGTCCTGGAAGCTAGAATCTGCATTTGCCAATGAAGGTTTCTTAGGCTACCAATACGAATGTCCAGGTTTGATTGAAGTCCCAACTGAGCAAGATCCTTCCAAATCTTATTGGGTCATGTTTATTTCTATCAACCCAGGTGCACCTGCTGGCGGTTCCTTCAACCAATATTTTGTTGGATCCTTCAATGGTACTCATTTTGAAGCGTTTGACAATCAATCTAGAGTGGTAGATTTTGGTAAGGACTACTATGCCTTGCAAACTTTCTTCAACACTGACCCAACCTACGGTTCAGCATTAGGTATTGCCTGGGCTTCAAACTGGGAGTACAGTGCCTTTGTCCCAACTAACCCATGGAGATCATCCATGTCTTTGGTCCGCAAGTTTTCTTTGAACACTGAATATCAAGCTAATCCAGAGACTGAATTGATCAATTTGAAAGCCGAACCAATATTGAACATTAGTAATGCTGGTCCCTGGTCTCGTTTTGCTACTAACACAACTCTAACTAAGGCCAATTCTTACAATGTCGATTTGAGCAACTCGACTGGTACCCTAGAGTTTGAGTTGGTTTACGCTGTTAACACCACACAAACCATATCCAAATCCGTCTTTGCCGACTTATCACTTTGGTTCAAGGGTTTAGAAGATCCTGAAGAATATTTGAGAATGGGTTTTGAAGTCAGTGCTTCTTCCTTCTTTTTGGACCGTGGTAACTCTAAGGTCAAGTTTGTCAAGGAGAACCCATATTTCACAAACAGAATGTCTGTCAACAACCAACCATTCAAGTCTGAGAACGACCTAAGTTACTATAAAGTGTACGGCCTACTGGATCAAAACATCTTGGAATTGTACTTCAACGATGGAGATGTGGTTTCTACAAATACCTACTTCATGACCACCGGTAACGCTCTAGGATCTGTGAACATGACCACTGGTGTCGATAATTTGTTCTACATTGACAAGTTCCAAGTAAGGGAAGTAAAATAGTTGGACTTCTTCGCCAGAGGTTTGGTCAAGTCTCCAATCAAGGTTGTCGGCTTGTCTACCTTGCCAGAAATTTACGAAAAGATGGAAAAGGGTCAAATCGTTGGTAGATACGTTGTTGACACTTCTAAATAAGCGAATTTCTTATGATTTATGATTTTTATTATTAAATAAGTTATAAAAAAAATAAGTGTATACAAATTTTAAAGTGACTCTTAGGTTTTAAAACGAAAATTCTTATTCTTGAGTAACTCTTTCCTGTAGGTCAGGTTGCTTTCTCAGGTATAGCATGAGGTCGCTCTTATTGACCACACCTCTACCGG

**(B)**
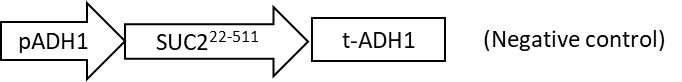


> ADH1 promoter-SUC2^22-511^ (without Signal peptide)-ADH1 terminator

ATCCTTTTGTTGTTTCCGGGTGTACAATATGGACTTCCTCTTTTCTGGCAACCAAACCCATACATCGGGATTCCTATAATACCTTCGTTGGTCTCCCTAACATGTAGGTGGCGGAGGGGAGATATACAATAGAACAGATACCAGACAAGACATAATGGGCTAAACAAGACTACACCAATTACACTGCCTCATTGATGGTGGTACATAACGAACTAATACTGTAGCCCTAGACTTGATAGCCATCATCATATCGAAGTTTCACTACCCTTTTTCCATTTGCCATCTATTGAAGTAATAATAGGCGCATGCAACTTCTTTTCTTTTTTTTTCTTTTCTCTCTCCCCCGTTGTTGTCTCACCATATCCGCAATGACAAAAAAATGATGGAAGACACTAAAGGAAAAAATTAACGACAAAGACAGCACCAACAGATGTCGTTGTTCCAGAGCTGATGAGGGGTATCTCGAAGCACACGAAACTTTTTCCTTCCTTCATTCACGCACACTACTCTCTAATGAGCAACGGTATACGGCCTTCCTTCCAGTTACTTGAATTTGAAATAAAAAAAAGTTTGCTGTCTTGCTATCAAGTATAAATAGACCTGCAATTATTAATCTTTTGTTTCCTCGTCATTGTTCTCGTTCCCTTTCTTCCTTGTTTCTTTTTCTGCACAATATTTCAAGCTATACCAAGCATACAATCAACTCCAGAATTCAGCTTTGCAAAGACAAACGAAACTAGCGATAGACCTTTGGTCCACTTCACACCCAACAAGGGCTGGATGAATGACCCAAATGGGTTGTGGTACGATGAAAAAGATGCCAAATGGCATCTGTACTTTCAATACAACCCAAATGACACCGTATGGGGTACGCCATTGTTTTGGGGCCATGCTACTTCCGATGATTTGACTAATTGGGAAGATCAACCCATTGCTATCGCTCCCAAGCGTAACGATTCAGGTGCTTTCTCTGGCTCCATGGTGGTTGATTACAACAACACGAGTGGGTTTTTCAATGATACTATTGATCCAAGACAAAGATGCGTTGCGATTTGGACTTATAACACTCCTGAAAGTGAAGAGCAATACATTAGCTATTCTCTTGATGGTGGTTACACTTTTACTGAATACCAAAAGAACCCTGTTTTAGCTGCCAACTCCACTCAATTCAGAGATCCAAAGGTGTTCTGGTATGAACCTTCTCAAAAATGGATTATGACGGCTGCCAAATCACAAGACTACAAAATTGAAATTTACTCCTCTGATGACTTGAAGTCCTGGAAGCTAGAATCTGCATTTGCCAATGAAGGTTTCTTAGGCTACCAATACGAATGTCCAGGTTTGATTGAAGTCCCAACTGAGCAAGATCCTTCCAAATCTTATTGGGTCATGTTTATTTCTATCAACCCAGGTGCACCTGCTGGCGGTTCCTTCAACCAATATTTTGTTGGATCCTTCAATGGTACTCATTTTGAAGCGTTTGACAATCAATCTAGAGTGGTAGATTTTGGTAAGGACTACTATGCCTTGCAAACTTTCTTCAACACTGACCCAACCTACGGTTCAGCATTAGGTATTGCCTGGGCTTCAAACTGGGAGTACAGTGCCTTTGTCCCAACTAACCCATGGAGATCATCCATGTCTTTGGTCCGCAAGTTTTCTTTGAACACTGAATATCAAGCTAATCCAGAGACTGAATTGATCAATTTGAAAGCCGAACCAATATTGAACATTAGTAATGCTGGTCCCTGGTCTCGTTTTGCTACTAACACAACTCTAACTAAGGCCAATTCTTACAATGTCGATTTGAGCAACTCGACTGGTACCCTAGAGTTTGAGTTGGTTTACGCTGTTAACACCACACAAACCATATCCAAATCCGTCTTTGCCGACTTATCACTTTGGTTCAAGGGTTTAGAAGATCCTGAAGAATATTTGAGAATGGGTTTTGAAGTCAGTGCTTCTTCCTTCTTTTTGGACCGTGGTAACTCTAAGGTCAAGTTTGTCAAGGAGAACCCATATTTCACAAACAGAATGTCTGTCAACAACCAACCATTCAAGTCTGAGAACGACCTAAGTTACTATAAAGTGTACGGCCTACTGGATCAAAACATCTTGGAATTGTACTTCAACGATGGAGATGTGGTTTCTACAAATACCTACTTCATGACCACCGGTAACGCTCTAGGATCTGTGAACATGACCACTGGTGTCGATAATTTGTTCTACATTGACAAGTTCCAAGTAAGGGAAGTAAAATAGTTGGACTTCTTCGCCAGAGGTTTGGTCAAGTCTCCAATCAAGGTTGTCGGCTTGTCTACCTTGCCAGAAATTTACGAAAAGATGGAAAAGGGTCAAATCGTTGGTAGATACGTTGTTGACACTTCTAAATAAGCGAATTTCTTATGATTTATGATTTTTATTATTAAATAAGTTATAAAAAAAATAAGTGTATACAAATTTTAAAGTGACTCTTAGGTTTTAAAACGAAAATTCTTATTCTTGAGTAACTCTTTCCTGTAGGTCAGGTTGCTTTCTCAGGTATAGCATGAGGTCGCTCTTATTGACCACACCTCTACCGG

**(C)**
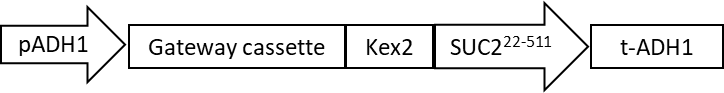


> ADH1 promoter-Gateway cassette-Kex2-SUC2^22-511^ (without Signal peptide)-ADH1 terminator

ATCCTTTTGTTGTTTCCGGGTGTACAATATGGACTTCCTCTTTTCTGGCAACCAAACCCATACATCGGGATTCCTATAATACCTTCGTTGGTCTCCCTAACATGTAGGTGGCGGAGGGGAGATATACAATAGAACAGATACCAGACAAGACATAATGGGCTAAACAAGACTACACCAATTACACTGCCTCATTGATGGTGGTACATAACGAACTAATACTGTAGCCCTAGACTTGATAGCCATCATCATATCGAAGTTTCACTACCCTTTTTCCATTTGCCATCTATTGAAGTAATAATAGGCGCATGCAACTTCTTTTCTTTTTTTTTCTTTTCTCTCTCCCCCGTTGTTGTCTCACCATATCCGCAATGACAAAAAAAATGATGGAAGACACTAAAGGAAAAAATTAACGACAAAGACAGCACCAACAGATGTCGTTGTTCCAGAGCTGATGAGGGGTATCTCGAAGCACACGAAACTTTTTCCTTCCTTCATTCACGCACACTACTCTCTAATGAGCAACGGTATACGGCCTTCCTTCCAGTTACTTGAATTTGAAATAAAAAAAAGTTTGCTGTCTTGCTATCAAGTATAAATAGACCTGCAATTATTAATCTTTTGTTTCCTCGTCATTGTTCTCGTTCCCTTTCTTCCTTGTTTCTTTTTCTGCACAATATTTCAAGCTATACCAAGCATACAATCAACTCCAGAATTCACAAGTTTGTACAAAAAAGCAGAACGAGAAACGTAAAATGATATAAATATCAATATATTAAATTAGATTTTGCATAAAAAACAGACTACATAATACTGTAAAACACAACATATCCAGTCACTATGAATCAACTACTTAGATGGTATTAGTGACCTGTAGTCGACCGACAGCCTTCCAAATGTTCTTCGGGTGATGCTGCCAACTTAGTCGACCGACAGCCTTCCAAATGTTCTTCTCAAACGGAATCGTCGTATCCAGCCTACTCGCTATTGTCCTCAATGCCGTATTAAATCATAAAAAGAAATAAGAAAAAGAGGTGCGAGCCTCTTTTTTGTGTGACAAAATAAAAACATCTACCTATTCATATACGCTAGTGTCATAGTCCTGAAAATCATCTGCATCAAGAACAATTTCACAACTCTTATACTTTTCTCTTACAAGTCGTTCGGCTTCATCTGGATTTTCAGCCTCTATACTTACTAAACGTGATAAAGTTTCTGTAATTTCTACTGTATCGACCTGCAGACTGGCTGTGTATAAGGGAGCCTGACATTTATATTCCCCAGAACATCAGGTTAATGGCGTTTTTGATGTCATTTTCGCGGTGGCTGAGATCAGCCACTTCTTCCCCGATAACGGAGACCGGCACACTGGCCATATCGGTGGTCATCATGCGCCAGCTTTCATCCCCGATATGCACCACCGGGTAAAGTTCACGGGAGACTTTATCTGACAGCAGACGTGCACTGGCCAGGGGGATCACCATCCGTCGCCCGGGCGTGTCAATAATATCACTCTGTACATCCACAAACAGACGATAACGGCTCTCTCTTTTATAGGTGTAAACCTTAAACTGCATTTCACCAGTCCCTGTTCTCGTCAGCAAAAGAGCCGTTCATTTCAATAAACCGGGCGACCTCAGCCATCCCTTCCTGATTTTCCGCTTTCCAGCGTTCGGCACGCAGACGACGGGCTTCATTCTGCATGGTTGTGCTTACCAGACCGGAGATATTGACATCATATATGCCTTGAGCAACTGATAGCTGTCGCTGTCAACTGTCACTGTAATACGCTGCTTCATAGCACACCTCTTTTTGACATACTTCGGGTATACATATCAGTATATATTCTTATACCGCAAAAATCAGCGCGCAAATACGCATACTGTTATCTGGCTTTTAGTAAGCCGGATCCACGCGATTACGCCCCGCCCTGCCACTCATCGCAGTACTGTTGTAATTCATTAAGCATTCTGCCGACATGGAAGCCATCACAGACGGCATGATGAACCTGAATCGCCAGCGGCATCAGCACCTTGTCGCCTTGCGTATAATATTTGCCCATGGTGAAAACGGGGGCGAAGAAGTTGTCCATATTGGCCACGTTTAAATCAAAACTGGTGAAACTCACCCAGGGATTGGCTGAGACGAAAAACATATTCTCAATAAACCCTTTAGGGAAATAGGCCAGGTTTTCACCGTAACACGCCACATCTTGCGAATATATGTGTAGAAACTGCCGGAAATCGTCGTGGTATTCACTCCAGAGCGATGAAAACGTTTCAGTTTGCTCATGGAAAACGGTGTAACAAGGGTGAACACTATCCCATATCACCAGCTCACCGTCTTTCATTGCCATACGGAATTCCGGATGAGCATTCATCAGGCGGGCAAGAATGTGAATAAAGGCCGGATAAAACTTGTGCTTATTTTTCTTTACGGTCTTTAAAAAGGCCGTAATATCCAGCTGAACGGTCTGGTTATAGGTACATTGAGCAACTGACTGAAATGCCTCAAAATGTTCTTTACGATGCCATTGGGATATATCAACGGTGGTATATCCAGTGATTTTTTTCTCCATTTTAGCTTCCTTAGCTCCTGAAAATCTCGATAACTCAAAAAATACGCCCGGTAGTGATCTTATTTCATTATGGTGAAAGTTGGAACCTCTTACGTGCCGATCAACGTCTCATTTTCGCCAAAAGTTGGCCCAGGGCTTCCCGGTATCAACAGGGACACCAGGATTTATTTATTCTGCGAAGTGATCTTCCGTCACAGGTATTTATTCGGCGCAAAGTGCGTCGGGTGATGCTGCCAACTTAGTCGACTACAGGTCACTAATACCATCTAAGTAGTTGATTCATAGTGACTGGATATGTTGTGTTTTACAGTATTATGTAGTCTGTTTTTTATGCAAAATCTAATTTAATATATTGATATTTATATCATTTTACGTTTCTCGTTCAGCTTTCTTGTACAAAGTGGTTGATGGGTACCCATACGACGTACCAGATTACGCTTCTCATGGTTCTTTGGATAAAAGAGAGGCTGAAGCTTGGGGATCTACAAACGAAACTAGCGATAGACCTTTGGTCCACTTCACACCCAACAAGGGCTGGATGAATGACCCAAATGGGTTGTGGTACGATGAAAAAGATGCCAAATGGCATCTGTACTTTCAATACAACCCAAATGACACCGTATGGGGTACGCCATTGTTTTGGGGCCATGCTACTTCCGATGATTTGACTAATTGGGAAGATCAACCCATTGCTATCGCTCCCAAGCGTAACGATTCAGGTGCTTTCTCTGGCTCCATGGTGGTTGATTACAACAACACGAGTGGGTTTTTCAATGATACTATTGATCCAAGACAAAGATGCGTTGCGATTTGGACTTATAACACTCCTGAAAGTGAAGAGCAATACATTAGCTATTCTCTTGATGGTGGTTACACTTTTACTGAATACCAAAAGAACCCTGTTTTAGCTGCCAACTCCACTCAATTCAGAGATCCAAAGGTGTTCTGGTATGAACCTTCTCAAAAATGGATTATGACGGCTGCCAAATCACAAGACTACAAAATTGAAATTTACTCCTCTGATGACTTGAAGTCCTGGAAGCTAGAATCTGCATTTGCCAATGAAGGTTTCTTAGGCTACCAATACGAATGTCCAGGTTTGATTGAAGTCCCAACTGAGCAAGATCCTTCCAAATCTTATTGGGTCATGTTTATTTCTATCAACCCAGGTGCACCTGCTGGCGGTTCCTTCAACCAATATTTTGTTGGATCCTTCAATGGTACTCATTTTGAAGCGTTTGACAATCAATCTAGAGTGGTAGATTTTGGTAAGGACTACTATGCCTTGCAAACTTTCTTCAACACTGACCCAACCTACGGTTCAGCATTAGGTATTGCCTGGGCTTCAAACTGGGAGTACAGTGCCTTTGTCCCAACTAACCCATGGAGATCATCCATGTCTTTGGTCCGCAAGTTTTCTTTGAACACTGAATATCAAGCTAATCCAGAGACTGAATTGATCAATTTGAAAGCCGAACCAATATTGAACATTAGTAATGCTGGTCCCTGGTCTCGTTTTGCTACTAACACAACTCTAACTAAGGCCAATTCTTACAATGTCGATTTGAGCAACTCGACTGGTACCCTAGAGTTTGAGTTGGTTTACGCTGTTAACACCACACAAACCATATCCAAATCCGTCTTTGCCGACTTATCACTTTGGTTCAAGGGTTTAGAAGATCCTGAAGAATATTTGAGAATGGGTTTTGAAGTCAGTGCTTCTTCCTTCTTTTTGGACCGTGGTAACTCTAAGGTCAAGTTTGTCAAGGAGAACCCATATTTCACAAACAGAATGTCTGTCAACAACCAACCATTCAAGTCTGAGAACGACCTAAGTTACTATAAAGTGTACGGCCTACTGGATCAAAACATCTTGGAATTGTACTTCAACGATGGAGATGTGGTTTCTACAAATACCTACTTCATGACCACCGGTAACGCTCTAGGATCTGTGAACATGACCACTGGTGTCGATAATTTGTTCTACATTGACAAGTTCCAAGTAAGGGAAGTAAAATAGTTGGACTTCTTCGCCAGAGGTTTGGTCAAGTCTCCAATCAAGGTTGTCGGCTTGTCTACCTTGCCAGAAATTTACGAAAAGATGGAAAAGGGTCAAATCGTTGGTAGATACGTTGTTGACACTTCTAAATAAGCGAATTTCTTATGATTTATGATTTTTATTATTAAATAAGTTATAAAAAAAATAAGTGTATACAAATTTTAAAGTGACTCTTAGGTTTTAAAACGAAAATTCTTATTCTTGAGTAACTCTTTCCTGTAGGTCAGGTTGCTTTCTCAGGTATAGCATGAGGTCGCTCTTATTGACCACACCTCTACCGG

**Supplemental Figure S2** Sequence of the yeast expression vectors for secretion analysis: **(A)** full length SUC2 with signal peptide (positive control), **(B)** SUC2 without signal peptide (negative control), **(C)** Vector for testing TaSSP secretion, including the gateway reading frame cassette and Kex2 cleavage site. The pGADT7 plasmid (Clontech, USA) was used as a backbone of the yeast expression vectors.

**Supplemental Figure S3** Relative expression of *TaSSP6* (**A**) and *TaSSP7* (**B**) in wheat leaves inoculated with *Z. tritici* isolate ‘Cork Cordiale 4. Gene expression for each sample was calculated as a percentage of the relative gene expression of the target gene (compared to the reference genes) in cv. Gallant at 4 dpi, treated with Tween 20 (control plants). qRT-PCR primers were designed to amplify all homoeologues of both *TaSSP* genes. Expression of *TaSSP* genes was induced by *Z. tritici* but not significantly so.

**Supplemental Figure S4** Development of pycnidia on wheat leaves treated with *Z. tritici* in plants that had silenced *TaSSP6* or *TaSSP7*. Silencing of both genes significantly increased the susceptibility to *Z. tritici* of the wheat cv. Stigg.

Supplemental Figure S5. ZtSSPs induced cell death in N. benthamiana leaves. The candidate ZtSSPs and/or interacted TaSSPs were expressed in leaves of *N. benthamiana* by *Agrobacterium*-mediated expression. (A) Three ZtSSPs (Zt06, Zt11 and Zt19) induced cell death phenotypes. (B-C) TaSSP6 and TaSSP7 did not induce cell death alone, and the co-expression of ZtSSP-TaSSP combinations did not alter the cell death phenotype in *N. benthamiana* leaves as compared to the ZtSSP proteins alone. GFP were expressed as negative control. Leaves photographed at 7 days post infiltration (dpi). Six leaves from 3 independent plants were used in this assay.

**Supplemental Table S1** Primer sets used in this study

| Primers | Primer sequence (5' to 3' | Application |
| --- | --- | --- |
| POP-IN-U2-F | CTCAGAGAAACAAGCAAAACAAAAAGCTTTTCTTTTCACTAACGTATATGGCATCAGAGCAGATTGTACTG | Yeast SUC2 gene knocked out |
| POP-IN-D2-R | TTTAGAATGGCTTTTGAAAAAAATAAAAAAGACAATAAGTTTTATAACCTGGTATTTCACACCGCATAGG | Yeast SUC2 gene knocked out |
| Pop-Trp-U2-F | CTCAGAGAAACAAGCAAAACAAAAAGCTTTTCTTTTCACTAACGTATATGGGCATCAGAGCAGATTGTAC | Yeast SUC2 gene knocked out |
| Pop-Trp-D2-R | TTTAGAATGGCTTTTGAAAAAAATAAAAAAGACAATAAGTTTTATAACCTCCTGATGCGGTATTTTCTCCT | Yeast SUC2 gene knocked out |
| TaSSP6-5utr-F | ACTAGAAGCACCGGAGAAGA | Cloning full length TaSSP6 |
| TaSSP6-3utr-R | GTAGAGTTGTACGCACACGTAG | Cloning full length TaSSP6 |
| TaSSP7-5utr-F | CCAATCTTCGAGAACGGTACAA | Cloning full length TaSSP7 |
| TaSSP7-3utr-R | GAGACACACTGCACGACATTA | Cloning full length TaSSP7 |
| AttB-TaSSP6-F | GGAGATAGAACCATGGCGTCCAAGGGTCTTCTTG | Cloning TaSSP7 into pDONR207 |
| AttB-dSP-TaSSP6-F | GGAGATAGAACCATGGAGCAAACTCAGGCCAAG | Cloning TaSSP6 without signal peptide into pDONR207 |
| AttB-TaSSP6-R | CAAGAAAGCTGGGTCGTTGCGGACCTCCGCGCGGTAC | Cloning TaSSP7 into pDONR207 |
| AttB-TaSSP6-Stop-R | CAAGAAAGCTGGGTCTCAGTTGCGGACCTCCGCGC | Cloning TaSSP7 into pDONR207 |
| qRT-TaSSP6-F1 | GTCCAAGGGTCTTCTTGTGTT | qPCR of TaSSP6 |
| qRT-TaSSP6-R1 | GGCTTTCTTCTCCTCCTTCTTG | qPCR of TaSSP6 |
| TaSSP6-VIGS1-F1 | GCCTTAATTAAAGCAAACTCAGGCCAAGAA | Cloning VIGS construct BSMV:TaSSP6-V1 |
| TaSSP6-VIGS1-R1 | ATAAGAATGCGGCCGCTCCTCCACCGTGTCCTC | Cloning VIGS construct BSMV:TaSSP6-V1 |
| TaSSP6-VIGS1-F2 | GCCTTAATTAAGAGGAGGCGGCGGCTAC | Cloning VIGS construct BSMV:TaSSP6-V1 |
| TaSSP6-VIGS2-R2 | ATAAGAATGCGGCCGCGTTGCGGACCTCCGC | Cloning VIGS construct BSMV:TaSSP6-V1 |
| AttB-TaSSP7-F | GGAGATAGAACCATGGCCGTCATGGCTTTCG | Cloning TaSSP7 into pDONR207 |
| AttB-dSP-TaSSP7-F | GGAGATAGAACCATGGTTACTGATGCGTCGGCTC | Cloning TaSSP7 without signal peptide into pDONR207 |
| AttB-TaSSP7-R | CAAGAAAGCTGGGTCATCTTCGGAGGGTTTTGCGA | Cloning TaSSP7 into pDONR207 |
| AttB-TaSSP7-Stop-R | CAAGAAAGCTGGGTCTCAATCTTCGGAGGGTTTTGC | Cloning TaSSP7 into pDONR207 |
| TaGAPDH for | CCTTCCGTGTTCCCACTGTTG | Control DNA contamination in RNA samples |
| TaGAPDH rev | ATGCCCTTGAGGTTTCCCTC | Control DNA contamination in RNA samples |
| Alpha tubulin F | ATCTCCAACTCCACCAGTGT | Housekeeping reference genes for qRT-PCR |
| Alpha tubulin R | TCATCGCCCTCATCACCGTC | Housekeeping reference genes for qRT-PCR |
| Glyceraldehyde phosphate dehydrogenase 2 F | TCACCACCGACTACATGA | Housekeeping reference genes for qRT-PCR |
| Glyceraldehyde phosphate dehydrogenase 2 R | ACAGCAACCTCCTTCTCA | Housekeeping reference genes for qRT-PCR |
| qRT-TaSSP7-F2 | TGGCTTTCGCTAATGGTCTC | qPCR of TaSSP7 |
| qRT-TaSSP7-R2 | ACGCATCAGTAACTGCCATC | qPCR of TaSSP7 |
| TaSSP7-VIGS1-F1 | GCCTTAATTAACGGCTCGGCTTTTGCAG | Cloning VIGS construct BSMV:TaSSP7-V1 |
| TaSSP7- VIGS1-R1 | ATAAGAATGCGGCCGCGCTGCAGAGGCAATCCTCA | Cloning VIGS construct BSMV:TaSSP7-V1 |
| TaSSP7- VIGS2-F2 | GCCTTAATTAAGGAGTCGCAAAACCCTCC | Cloning VIGS construct BSMV:TaSSP7-V2 |
| TaSSP7- VIGS2-R2 | ATAAGAATGCGGCCGCGAGGGTTTTGCGACTCCA | Cloning VIGS construct BSMV:TaSSP7-V2 |
| AttB-TaTRG7-F | GGAGATAGAACCATGAGCTCCTCGGACGACAC | Cloning TaTRG7 into pDONR207 |
| AttB-TaTRG7-R | CAAGAAAGCTGGGTCGCCAATGCGGGGGATCTC | Cloning TaTRG7 into pDONR207 |
| AttB-TaTRG7-Stop-R | CAAGAAAGCTGGGTCCTAGCCAATGCGGGGGATCTC | Cloning TaTRG7 into pDONR207 |
| attB1 | GGGGACAAGTTTGTACAAAAAAGCAGGCTTCGAAGGAGATAGAACCATG | attB extension for subcloning into pDONR207 |
| attB2 | GGGGACCACTTTGTACAAGAAAGCTGGGTC | attB extension for subcloning into pDONR207 |
| P45-BamH1-Zt11 | GCGGATCCCAAATACTGCGCCGG | Cloning Zt11 into pET45b+ expression vector |
| P45-Xho1-Zt11 | CCGCTCGAGTCAAGTTCCTGGGCA | Cloning Zt11 into pET45b+ expression vector |

**Supplemental Table 2** List of twenty-seven *Z. tritici* candidate small, secreted proteins. The sequence could be found in Joint Genome Institute, The Fungal Genomics Resource (<https://mycocosm.jgi.doe.gov/Mycgr3/Mycgr3.home.html>)

| Zt code | JGI Protein ID |
| --- | --- |
| ZG01 | Mycgr3G67799 |
| ZG02 | [Mycgr3G73448](http://fungi.ensembl.org/Zymoseptoria_tritici/Transcript/Summary?db=core;g=Mycgr3G73448;r=6:2111850-2112880;t=Mycgr3T73448) |
| ZG03 | [Mycgr3G79783](http://fungi.ensembl.org/Zymoseptoria_tritici/Transcript/Summary?db=core;g=Mycgr3G79783;r=3:513058-513898;t=Mycgr3T79783) |
| ZG04 | [Mycgr3G81079](http://fungi.ensembl.org/Zymoseptoria_tritici/Transcript/Summary?db=core;g=Mycgr3G81079;r=6:572649-573552;t=Mycgr3T81079) |
| ZG05 | [Mycgr3G100649](http://fungi.ensembl.org/Zymoseptoria_tritici/Transcript/Summary?db=core;g=Mycgr3G100649;r=7:263827-264330;t=Mycgr3T100649) |
| ZG06 | [Mycgr3G101652](http://fungi.ensembl.org/Zymoseptoria_tritici/Transcript/Summary?db=core;g=Mycgr3G101652;r=11:1326756-1327372;t=Mycgr3T101652) |
| ZG07 | [Mycgr3G102617](http://fungi.ensembl.org/Zymoseptoria_tritici/Transcript/Summary?db=core;g=Mycgr3G102617;r=1:3652349-3653208;t=Mycgr3T102617) |
| ZG08 | [Mycgr3G103091](http://fungi.ensembl.org/Zymoseptoria_tritici/Transcript/Summary?db=core;g=Mycgr3G103091;r=2:828240-828840;t=Mycgr3T103091) |
| ZG09 | [Mycgr3G103900](http://fungi.ensembl.org/Zymoseptoria_tritici/Transcript/Summary?db=core;g=Mycgr3G103900;r=3:2843555-2844780;t=Mycgr3T103900) |
| ZG10 | [Mycgr3G104404](http://fungi.ensembl.org/Zymoseptoria_tritici/Transcript/Summary?db=core;g=Mycgr3G104404;r=5:684945-685845;t=Mycgr3T104404) |
| ZG11 | [Mycgr3G104444](http://fungi.ensembl.org/Zymoseptoria_tritici/Transcript/Summary?db=core;g=Mycgr3G104444;r=5:989972-990541;t=Mycgr3T104444) |
| ZG12 | [Mycgr3G104697](http://fungi.ensembl.org/Zymoseptoria_tritici/Transcript/Summary?db=core;g=Mycgr3G104697;r=5:2792882-2793612;t=Mycgr3T104697) |
| ZG13 | Mycgr3G104794 |
| ZG14 | [Mycgr3G105182](http://fungi.ensembl.org/Zymoseptoria_tritici/Transcript/Summary?db=core;g=Mycgr3G105182;r=7:1005513-1006183;t=Mycgr3T105182) |
| ZG15 | [Mycgr3G105223](http://fungi.ensembl.org/Zymoseptoria_tritici/Transcript/Summary?db=core;g=Mycgr3G105223;r=7:1219399-1220237;t=Mycgr3T105223) |
| ZG16 | [Mycgr3G105659](http://fungi.ensembl.org/Zymoseptoria_tritici/Transcript/Summary?db=core;g=Mycgr3G105659;r=9:381747-382480;t=Mycgr3T105659) |
| ZG17 | [Mycgr3G105826](http://fungi.ensembl.org/Zymoseptoria_tritici/Transcript/Summary?db=core;g=Mycgr3G105826;r=10:107365-108215;t=Mycgr3T105826) |
| ZG18 | [Mycgr3G107286](http://fungi.ensembl.org/Zymoseptoria_tritici/Transcript/Summary?db=core;g=Mycgr3G107286;r=1:3158807-3159576;t=Mycgr3T107286) |
| ZG19 | [Mycgr3G108482](http://fungi.ensembl.org/Zymoseptoria_tritici/Transcript/Summary?db=core;g=Mycgr3G108482;r=3:780398-781083;t=Mycgr3T108482) |
| ZG20 | [Mycgr3G110220](http://fungi.ensembl.org/Zymoseptoria_tritici/Transcript/Summary?db=core;g=Mycgr3G110220;r=7:1373544-1374635;t=Mycgr3T110220) |
| ZG21 | [Mycgr3G111008](http://fungi.ensembl.org/Zymoseptoria_tritici/Transcript/Summary?db=core;g=Mycgr3G111008;r=10:618329-619090;t=Mycgr3T111008) |
| ZG22 | [Mycgr3G111382](http://fungi.ensembl.org/Zymoseptoria_tritici/Transcript/Summary?db=core;g=Mycgr3G111382;r=12:121814-122850;t=Mycgr3T111382) |
| ZG23 | [Mycgr3G111505](http://fungi.ensembl.org/Zymoseptoria_tritici/Transcript/Summary?db=core;g=Mycgr3G111505;r=12:898626-899362;t=Mycgr3T111505) |
| ZG24 | [Mycgr3G99161](http://fungi.ensembl.org/Zymoseptoria_tritici/Transcript/Summary?db=core;g=Mycgr3G99161;r=2:2101447-2102282;t=Mycgr3T99161) |
| ZG25 | [Mycgr3G105896](http://fungi.ensembl.org/Zymoseptoria_tritici/Transcript/Summary?db=core;g=Mycgr3G105896;r=10:542310-543174;t=Mycgr3T105896) |
| ZG26 | [Mycgr3G106445](http://fungi.ensembl.org/Zymoseptoria_tritici/Transcript/Summary?db=core;g=Mycgr3G106445;r=13:136802-137670;t=Mycgr3T106445) |
| ZG27 | [Mycgr3G108774](http://fungi.ensembl.org/Zymoseptoria_tritici/Transcript/Summary?db=core;g=Mycgr3G108774;r=3:2648032-2649040;t=Mycgr3T108774) |
